# Semantic Recollection in Parkinson’s Disease: Functional Reconfiguration and MAPT Variants

**DOI:** 10.3389/fnagi.2021.727057

**Published:** 2021-09-20

**Authors:** Deborah L. Harrington, Qian Shen, Vida Sadeghi, Mingxiong Huang, Irene Litvan, Xiangyu Wei, Roland R. Lee

**Affiliations:** ^1^Research and Radiology Services, VA San Diego Healthcare System, San Diego, CA, United States; ^2^Department of Radiology, University of California, San Diego, La Jolla, CA, United States; ^3^Department of Neurosciences, University of California, San Diego, La Jolla, CA, United States

**Keywords:** Parkinson’s disease, semantic network, cognition, fMRI, functional connectivity, MAPT gene

## Abstract

Decline in semantic cognition in early stages of Parkinson’s disease (PD) is a leading risk factor for future dementia, yet the underlying neural mechanisms are not understood. The present study addressed this gap by investigating the functional connectivity of regions involved in semantic recollection. We further examined whether microtubule-associated protein tau (MAPT) risk variants, which may accelerate cognitive decline, altered the strength of regional functional connections. Cognitively normal PD and healthy elder controls underwent fMRI while performing a fame-discrimination task, which activates the semantic network. Analyses focused on disturbances in fame-modulated functional connectivity in PD for regions that govern semantic recollection and interrelated processes. Group differences were found in multiple connectivity features, which were reduced into principal components that reflected the strength of fame-modulated regional couplings with other brain areas. Despite the absence of group differences in semantic cognition, two aberrant connectivity patterns were uncovered in PD. One pattern was related to a loss in frontal, parietal, and temporal connection topologies that governed semantic recollection in older controls. Another pattern was characterized by functional reconfiguration, wherein frontal, parietal, temporal and caudate couplings were strengthened with areas that were not recruited by controls. Correlations between principal component scores and cognitive measures suggested that reconfigured frontal coupling topologies in PD supported compensatory routes for accessing semantic content, whereas reconfigured parietal, temporal, and caudate connection topologies were detrimental or unrelated to cognition. Increased tau transcription diminished recruitment of compensatory frontal topologies but amplified recruitment of parietal topologies that were unfavorable for cognition. Collectively, the findings provide a new understanding of early vulnerabilities in the functional architecture of regional connectivity during semantic recollection in cognitively normal PD. The findings also have implications for tracking cognitive progression and selecting patients who stand to benefit from therapeutic interventions.

## Introduction

In early stages of Parkinson’s disease (PD) cognitive decline is prominent in attention and executive functions, but memory, visuospatial cognition, and semantic cognition can also be affected (Muslimovic et al., 2005). The diversity in the cognitive domains affected suggests that patterns of neurodegeneration differ amongst people. The dual-syndrome hypothesis distinguishes between frontostriatal executive and posterior cortical visuospatial-mnemonic impairments in PD, which may be affected differently by dopaminergic, noradrenergic, and cholinergic loss (Kehagia et al., 2013; Gratwicke et al., 2015) and genetic factors that carry different prognostic significance (Lin and Wu, 2015). Temporoparietal neurodegeneration is particularly important as it may underly early changes in semantic cognition (e.g., measured by category fluency and naming) in PD, which is a leading risk factor for later mild cognitive impairment (MCI) (Hobson and Meara, 2015) and dementia (Williams-Gray et al., 2009; Evans et al., 2011; Compta et al., 2013; Williams-Gray et al., 2013). Yet the pathophysiological underpinnings of semantic cognition in PD have not been well delineated, especially before clinical symptoms manifest, which is vital since optimal interventions will depend on early detection.

Semantic memory stores a person’s knowledge about the world. It is deployed constantly to understand concepts and categories, recall familiar information, and recognize objects. Semantic knowledge is formed through experience-related inputs from sensorimotor, visual, and conceptual systems (Ralph et al., 2017). These systems communicate with the anterior temporal lobe, which integrates multimodal features that shape semantic representations. This hypothesis aligns with focal atrophy of the anterior temporal cortex in semantic dementia (Lambon Ralph and Patterson, 2008) and the semantic variant of primary progressive aphasia (Gorno-Tempini et al., 2011). Some models propose that the anterior temporal lobe is a semantic-selective hub that is responsible for stable representations of semantic knowledge (Ralph et al., 2017), whereas other regions control semantic access. Other models propose that there are many semantic hubs (Binder and Desai, 2011) as posterior temporoparietal convergence zones (e.g., angular gyrus) also shape semantic memory via integration of inputs from diverse networks (Bonner et al., 2013; Fairhall and Caramazza, 2013; Price et al., 2015; Binder et al., 2016). The process of remembering is multifaceted, involving partial reactivation of temporoparietal semantic networks (Danker and Anderson, 2010; Garcia et al., 2020), but also recruitment of frontal regions that supervise accesses to semantic knowledge (Binder and Desai, 2011; Chiou et al., 2018) as well as memory (parahippocampus, hippocampus) and retrieval systems (e.g., SMA, precuneus, posterior cingulate) (Cavanna and Trimble, 2006; Ranganath and Ritchey, 2012; Danker et al., 2017).

The multilayered nature of semantic recollection is germane to understanding common semantic cognition disturbances in PD such as word finding difficulties (Galtier et al., 2019). Deficits in various aspects of semantic cognition (e.g., object semantics, noun/verb generation, semantic fluency, word finding) in PD can be related to executive dysfunction (Bocanegra et al., 2015; Cousins and Grossman, 2017; Silveri et al., 2018), which aligns with the correlation between semantic fluency and frontal cortical thinning (Pereira et al., 2009, 2014). However, executive dysfunction in PD correlates with disturbances in object/noun processing, but not action/verb processing (Bocanegra et al., 2015), suggesting that executive influences on language processing depend upon the semantic category. This finding aligns with others who suggest that word finding difficulties are principally semantic in nature, owing to deficient processing of semantic content (Auclair-Ouellet et al., 2017). Indeed, semantic but not phonemic fluency performance in PD is associated with temporal cortex thinning (Pereira et al., 2009), which may render memories of semantic content less coherent. Thus, the mechanisms for semantic deficits in PD may vary, possibly due to individual differences in regional neuropathology, some of which may foreshadow the development of MCI and dementia. The neural bases for early changes in semantic cognition, however, have not been studied in cognitively normal PD using functional imaging.

Heterogeneity in semantic decline may also be partly related to genetic variants, which render certain brain systems more vulnerable to neurodegeneration. Microtubule-associated protein tau (MAPT) is a protein that forms pathological aggregates in several neurodegenerative diseases. The H1 haplotype promotes tau aggregation, which interacts with α-synuclein in Lewy body formation (Colom-Cadena et al., 2013a; Robakis et al., 2016; Smith et al., 2019). The H1/H1 genotype is thought to accelerate cognitive decline in early years of PD (Goris et al., 2007; Williams-Gray et al., 2009; Sampedro et al., 2018). Although regional vulnerabilities to the expression of MAPT are not well understood, PD H1 homozygotes show greater frontal and temporal-parietal atrophy (Sampedro et al., 2018) and decreased posterior cortex activation relative to PD H2 carriers (Nombela et al., 2014; Winder-Rhodes et al., 2015). However, MAPT effects on brain functioning during semantic recollection have not been studied in PD, nor have subhaplotypes of the H1 lineage, which encode tau transcription activity levels in PD (Compta et al., 2011).

The present study sought to address these gaps by investigating the neural mechanisms underlying semantic cognition in cognitively normal PD and healthy aging cohorts, which has not been previously studied. Participants underwent functional magnetic resonance imaging (fMRI) as they performed a fame-discrimination task, for which famous name recollection produces greater activation than unfamiliar names in the temporoparietal semantic network as well as executive and memory systems (Woodard et al., 2010; Rao et al., 2015). Since cognition arises from interactions amongst brain regions, fMRI analyses focused on identifying disturbances in fame-modulated functional connectivity for regions that govern semantic cognition and supporting processes. Abnormal functional connections were then correlated with measures of semantic cognition and interdependent cognitive functions to elucidate their behavioral relevance. To unravel heterogeneity in the pathophysiological underpinnings of semantic cognition in PD, we examined whether MAPT risk variants, which are thought to accelerate cognitive decline early in PD (Williams-Gray et al., 2009), altered the strength of regional functional connections. As greater tau expression in PD is linked to atrophy in temporal-parietal regions (Sampedro et al., 2018), which support semantic cognition (Binder and Desai, 2011), we predicted that greater tau expression in PD would correlate with more aberrant coupling strengths of posterior cortical areas of the semantic network.

## Materials and Methods

### Participants

The sample were 63 cognitively normal PD participants who met the PD United Kingdom Brain Bank Criteria and 43 healthy controls. Exclusion criteria included metal in the head, neurological diagnoses other than PD, psychiatric diagnoses, history of alcohol or substance abuse, positive MRI findings (e.g., infarcts, vascular disease), use of anticholinergics or cognitive medications, and complaints of cognitive deficits. PD volunteers with tremors or dyskinesias that might cause head motion were excluded. Volunteers were excluded if they met the Movement Disorders Society Level II criteria for PD-MCI (Litvan et al., 2012). MCI was defined as >1.5 standard deviations below the control group mean on at least two tests in single or different domains. There were six *de novo* patients, five patients taking dopamine agonist monotherapy, 26 taking levodopa monotherapy, and 26 taking levodopa combination therapy. Testing was conducted *on* medication therapy. The Institutional Review Board at the VA San Diego Healthcare System approved the study. All subjects signed written informed consent.

The groups did not differ in age, educational level, sex, handedness, or premorbid intelligence (Wechsler Test of Adult Reading) (Table 1). The PD group had significantly lower scores than controls on the long delay free recall tests of verbal and visual episodic memory (California Verbal Learning Test 2, CVLT-II; Brief Visuospatial Memory Test-Revised, BVMT-R) and visuospatial cognition (Judgment of Line Orientation; Hooper Visual Organization, HVOT). This indicates a decline at the group level in these functions, but individual patients did not exhibit clinically significant cognitive decline indicative of MCI.

**TABLE 1:** Demographic, clinical, genotypic, and cognitive characteristics.

	Parkinson’s (*n* = 63)	Control (*n* = 43)	*p*	η_*p*_^2^
Age (years)	65.3 (6.5)	64.1(8.5)	0.39	0.01
Education (years)	17.0 (2.1)	17.0 (2.1)	0.88	0.00
Sex (% females)	41.3%	44.2%	0.77	
Handedness (% right-handed)	84.1%	88.4%	0.54	
Wechsler Test of Adult Reading	44.4 (4.9)	45.6 (3.8)	0.22	0.02
Montreal Cognitive Assessment	27.0 (2.3)	27.6 (2.0)	0.15	0.03
Beck Depression Inventory	6.5 (5.6)	2.4 (3.5)	0.001	0.16
Disease duration (years)	4.7 (3.8)			
Levodopa dosage equivalence**^†^**	927 (654)			
UPDRS Part III	23.0 (11.4)			
Hoehn and Yahr stage1:2:3:4	12:48:2:1			
MAPT rs9468 H1/H1:H2	43:20	32:11	0.49	
MAPT rs242557 GG:A	30:33	13:30	0.07	
**Attention and working memory**				
Adaptive Digit Ordering	6.4 (1.8)	6.6 (2.2)	0.58	0.00
DKEFS Color + Word Naming	22.2 (7.3)	21.8 (4.5)	0.75	0.00
**Executive functioning (DKEFS)**				
Category Switching (accuracy)	13.5 (2.8)	13.3 (3.1)	0.79	0.00
Color-Word Inhibition/Switching**^§^**	63.8 (21.7)	58.1 (13.6)	0.13	0.02
DKEFS Letter Fluency^‡^	45.2 (12.0)	49.3 (12.6)	0.09	0.03
**Episodic memory**				
CVLT-II Long Delay Free Recall	9.1 (3.3)	11.3 (3.0)	0.001	0.11
BVMT-R Long Delay Free Recall	8.2 (2.6)	9.9 (1.9)	0.001	0.11
**Visuospatial processing**				
Judgment of Line Orientation	25.3 (2.8)	26.9 (2.7)	0.004	0.08
Hooper Visual Organization	25.4 (2.3)	27.3 (3.3)	0.001	0.10
**Semantic Language**				
Boston Naming	57.6 (2.6)	58.3 (1.7)	0.12	0.02
DKEFS Category Fluency^‡^	43.3 (8.7)	44.2 (9.1)	0.61	0.00
**Fame discrimination task**				
d’	3.6 (0.6)	3.3 (0.81)	0.34	0.01
% correct: famous names, unfamiliar names	0.90 (0.08) 0.96 (0.10)	0.89 (0.10) 0.96 (0.08)	0.69	0.00
Reaction time: famous names, unfamiliar names	1178 (242) 1268 (309)	1164 (215) 1298 (283)	0.32	0.01

*Tabled values are raw score means (standard deviations), except for genotypes, which are expressed as the frequency of different alleles. Group differences were tested using ANOVA and Pearson chi-square statistics (sex, handedness, and genotype).*

*^†^Levodopa dosage equivalence was calculated using the method of Tomlinson (Tomlinson et al., 2010). Data are based on 57 participants who were taking dopaminergic therapy.*

*^§^ An outlier was found for one PD participant, who had a raw score of 180. When this subject was removed from the data, tests for group differences remained non-significant [PD mean (SD) = 62.0 (15.9); *p* < 0.20, η_*p*_^2^ = 0.02].*

*^‡^Letters for the Letter Fluency test were F, A, and S. Categories for the Category Fluency test were animals and boy’s names.*

*BVMT-R, Brief Visuospatial Memory Test-Revised; CVLT-II, California Verbal Learning Test Version 2; DKEFS, Delis Kaplan Executive Function System; UPDRS, Unified Parkinson’s Disease Rating Scale.*

### Genotyping

Oragene-500 kits^1^ were used to collect whole saliva samples (2 mL). TaqMan assays were used for genotyping MAPT polymorphisms relevant to PD (Goris et al., 2007; Chen et al., 2016; Zhang et al., 2017). MAPT rs9468 tags the H1 and H2 haplotypes. The MAPT H1 subhaplotype rs242557 represents the intra-H1 variation in transcriptional activity, with the A allele associated with higher tau transcription levels than the G allele (Compta et al., 2011; Chen et al., 2017). Group differences in the distributions of allele types were non-significant (Chi-square: rs9468: *p* > 0.49; rs24557: *p* > 0.08; Supplementary Table 1).

### Imaging Protocols

Imaging was conducted on a GE MR750 Discovery 3 Tesla system equipped with a Nova Medical 32-channel head coil. Head motion was limited by foam pads inserted between the head and the coil. Visual stimuli were projected onto a screen and viewed through a mirror. Non-ferrous keypad devices interfaced with a computer recorded task performance for off-line analysis. High-resolution T1-weighted anatomical images were acquired (3D spoiled gradient-recalled at steady state, minimum full TE, 3.5 ms; TR, 2852 ms; TI, 1000 ms; 8° flip angle; 0.8-mm slices acquisition matrix = 512). For task-activated fMRI (tafMRI), a high spatial and temporal resolution multiband-protocol was used, which has greater sensitivity and specificity than conventional single-band echo-planar protocols (Tomasi et al., 2016). The protocol was a multiband accelerated gradient-echo planar imaging sequence with slice thickness = 2 mm, TR = 800 ms, TE = 35 ms, flip angle = 52°, acquisition matrix = 104, axial slices = 72, multiband factor = 8, echo spacing = 0.612 ms, band width = 4807.69 Hz/Px. The first two multiband factor repetitions (12.8 s) were removed to allow magnetization to stabilize to a steady state. Total time of the tafMRI run was 5 min and 57 s. To correct for geometric distortions in the data, a pair of gradient EPI sequences were acquired immediately before the tafMRI scan (anterior and posterior reversed gradients; TR = 8500 ms, TE = 70.6 ms, 2 mm isotropic voxels, flip angle = 90° and echo spacing = 0.612 ms).

### Semantic Memory Task

To probe for brain activity related to semantic processing, the famous name discrimination task was used as it is performed with high accuracy and low effort, thereby minimizing effects of executive dysfunction on semantic recollection (Rao et al., 2015). Famous name recollection is a sensitive preclinical marker of neurocognitive decline in longitudinal studies of healthy elders with normal cognition (Woodard et al., 2010; Seidenberg et al., 2013; Rao et al., 2015) and also distinguishes patients with semantic dementia from people with Alzheimer’s disease, who perform more poorly than normal aging adults (Snowden et al., 2004). Stimuli for the task were drawn from a pool of famous names (e.g., entertainers, politicians, athletes) from the 1990s with a high identification rate (>90%) (Douville et al., 2005) and unfamiliar names. The task contained 30 famous and 30 unfamiliar names, which were randomly presented. On each trial a name was visually presented for 3 s and the participant responded as quickly as possible, making a right index or middle finger key press if the name was famous or unfamiliar respectively. Intertrial intervals consisted of randomly jittered (2000--7200 ms) filler trials in which the participant fixated on a central crosshair. Randomized stimulus timing parameters were optimized using RSFgen from the Analysis of Functional NeuroImages (AFNI) software^2^. The dependent measures were (1) reaction time (RT) for correct trials (time from stimulus onset to a keypress), (2) percent correct, and (3) *d* prime (*d*’), a measure of sensitivity that adjusts for response biases (*d*’ = inverse of the standard normal cumulative distributions of hits – inverse of the standard normal cumulative distribution of false alarms).

### Image Analyses

To correct for geometric distortions in the data, a field map was computed from the pair of anterior and posterior reversed gradient sequences using AFNIto3d. The field map was applied to the tafMRI data using the FMRIB Software Library TOPUP program^3^. The standard processing pipeline included (1) volume registration to the first echo-planar volume and head motion correction (3dvolreg); (2) alignment to a skull-stripped anatomical T1-weighted structural image and warping to the MNI space; and (3) spatial smoothing using an isotropic Gaussian filter kernel with a full-width at half-maximum of 6 mm to minimize inter-subject variability. No group differences were found in framewise displacement [PD: 0.071 (0.034); Control: 0.065 (0.027); *F* = 1.0, *p* < 0.32, η_*p*_^2^ = 0.01]. Thus, procedures to limit head motion were effective.

#### Voxelwise Tests of Name Familiarity and Group Effects

AFNI 3dDeconvolve was used to estimate the hemodynamic response function (HRF) of each voxel using multiple linear regressions. The analysis pipeline included deconvolution of each subject’s time series for correct trials in each condition (famous and unfamiliar names) and 12 motion parameters (six translational/rotational axes and six motion derivatives). Each HRF was estimated relative to the baseline state (filler images). Incorrect trials were regressed out of the time series at each voxel. The contrast of interest compared the differences in the magnitude of the signal for famous names versus unfamiliar names. A mixed model ANOVA tested the effect of name familiarity and its interaction with group using AFNI 3dMVM. Monte Carlo simulations with 10,000 iterations (3dClustSim using the ACF method) computed the voxel-probability and minimum cluster-size threshold needed to obtain a familywise alpha. To test the main effect of condition (famous versus unfamiliar), a corrected alpha of *p* < 0.05 was obtained using a voxelwise probability of *p* < 0.0001 and a minimum cluster size of ≥18.2 voxels. To determine if the magnitude of activation between famous and unfamiliar names differed between PD and controls, ANOVAs tested for group by condition effects using a voxelwise probability of *p* < 0.005 and a minimum cluster size of ≥107 voxels to obtain a corrected alpha of *p* < 0.05.

#### Fame-Modulated Functional Connectivity Analyses (gPPI)

Hypotheses testing focused on whether group differences in the fame-modulated connectivity of a seed region of interest (ROI) with other brain areas depended on name familiarity (famous versus unfamiliar names). To this end, the generalized psychophysical interaction (gPPI) model as implemented in AFNI was used. The gPPI approach analyzes the physiological response of a ROI (i.e., hemodynamic response convolved blood-oxygen-level dependent signal) in terms of its context-dependent coupling with other brain regions (McLaren et al., 2012). This produced measures of fame-modulated functional connectivity between two or more regions. Selection of seed ROI for the gPPI analyses was theoretically and empirically driven by regions that showed significantly greater activation for famous than unfamiliar names in voxelwise analyses and were components of the temporal-parietal semantic network and interdependent systems (executive, memory, retrieval).

For the gPPI analyses, 12 mm diameter seeds that were placed in regions where peak activation was greater for famous than unfamiliar names. For small volume structures (i.e., parahippocampus, hippocampus, caudate), the seed encompassed all voxels showing greater activation for famous than unfamiliar names. The physiological variable was created by extracting the mean deconvolved times courses from a seed region for each subject. PPI interaction terms were computed as the cross product of the physiological variable and the task condition (i.e., famous names, unfamiliar names). Nuisance variables were error trials for the task condition and 12 motion regressors (six translational and rotational axes and their six temporal derivatives). This produced a first-level model with 14 nuisance variables and three regressors for each seed (one task condition, one interaction term, and the time course of one seed). The regression produced correlation maps for the time course in the seed ROI with the time course from all other brain voxels as a function of a task condition. Fisher *z* transforms were applied to the correlation maps. Second-level analyses tested the interaction of group with the name familiarity contrast from the first level analyses, as implemented by AFNI 3dMVM. Because spatial thresholds are biased against small volume structures, thresholds for the PPI analyses were derived separately for these ROI. A corrected alpha (*p* < 0.05) was obtained using a voxelwise probability of *p* < 0.005 and a minimum cluster size of 77 voxels for the cortex and 35 voxels for small-volume regions (10,000 simulations using the AFNI ACF method). The false discovery rate (FDR; *q* < 0.001) was applied to *corrected p*-values from the gPPI analyses to further adjust for analyses of multiple seeds.

### Statistical Analyses

#### Principal Component Analyses (PCA)

Features that showed group differences in fame-modulated functional connectivity in the gPPI analyses were condensed into components using PCA. As frontal, parietal, temporal and striatal areas govern different facets of semantic cognition (Binder and Desai, 2011) that are relevant to PD, PCA was conducted separately for features associated with these regions to characterize their functional interactions with the rest of the brain (i.e., topologies) and determine if their coupling strengths differed between the groups. An oblique rotation (Promax) implemented in SPSS 27 was applied. For each derived principal component (PC), a score was computed using the regression method in SPSS 27, which converts variables into *z*-scores, multiplies them by their pattern weight, and computes the weighted linear combination of the variables. PC scores therefore reflect the strength of regional fame-modulated couplings (famous > unfamiliar names) with other brain areas. Multivariate analyses of variance (MANOVA) tested whether sets of PC scores (multiple dependent variables) significantly differed between the groups. PC scores were used in subsequent analyses to test for their associations with cognitive and genetic variables.

#### Principal Component Score Relationships to Semantic Cognition and Other Processes

Stepwise multiple regression analyses were used to identify PCs (independent variables) that best accounted for individual differences in fame discrimination (*d*’), semantic fluency (Category Fluency), and confrontation naming (Boston Naming). In each group, regression analyses were performed separately for frontal, parietal, temporal, and striatal PC scores (FDR corrected; *q* ≤ 0.05). The same analyses tested for the relationships between PC scores and selected cognitive functions that can influence semantic processing (FDR corrected) including (1) executive functions (Letter Fluency and Color-Word Inhibition/Switching); (2) verbal/visual episodic memory (CVLT-II, BVMT-R); and (3) the HVOT, a measure of visual organization that requires object name recollection. As some neuropsychological variables correlated with age, analyses were performed on age adjusted residuals.

#### Component Score Associations With Genetic Variants

Multivariate analyses of variances tested for relationships between genes and sets of PC scores. Models tested for the main effect of gene and its interaction with group for each MAPT polymorphism.

## Results

### Fame Discrimination Performance

Average accuracy ranged between 89 to 96% correct for both groups (Table 1). Group and group by familiarity interactions were non-significant for RT (*p* > 0.86) and percent correct (*p* > 0.68). In both groups RTs were faster and accuracy was lower for famous than unfamiliar names [RT: *F* = 26.5, *p* < 0.00001, η_*p*_^2^ = 0.20; percent correct: *F* = 30.0, *p* < 3.003E-7; η_*p*_^2^ = 0.22]. No group differences were found for *d*’ (*p* > 0.34).

### Voxelwise Tests of Fame-Related Effects

**FIGURE 1:**
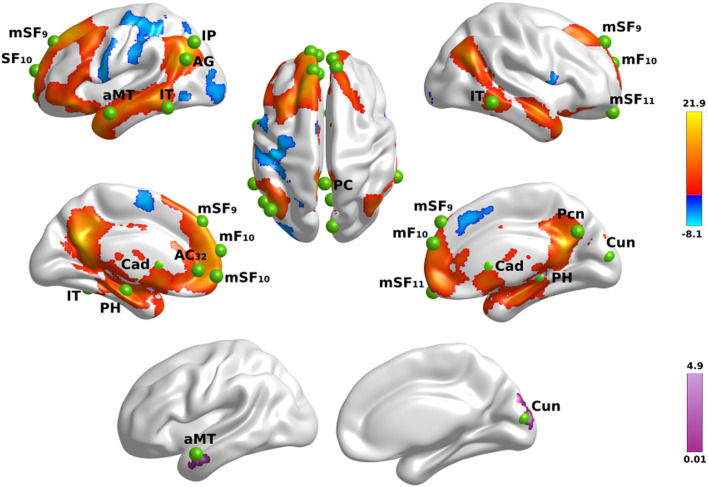
Name familiarity effects on brain activation. **Top rows** display left and right hemisphere regional activations from voxelwise tests of name familiarity in all participants. Warm colors designate activations that were greater for familiar than unfamiliar names and cool colors designate activations that were greater for unfamiliar than familiar names. The **bottom row** displays areas showing group differences in the effects of name familiarity. Color bars show the range of *F* values for significant effects, which are detailed in Supplementary Table 2. Green balls illustrate the locations of seed regions of interest, which were used in the gPPI analyses. Nineteen seeds were placed in areas showing significantly greater activation for famous than unfamiliar names (see Supplementary Table 3 for seed coordinates). Brodmann areas for frontal seeds are designated by subscripts. AC, anterior cingulate; AG, angular gyrus; aMT, anterior middle temporal; Cad, caudate; Cun, cuneus; IP, inferior parietal; IT, inferior temporal; mF, medial frontal; mSF, medial superior frontal; Pcn, precuneus; PC, posterior cingulate; PH, parahippocampus; SF, superior frontal.

First, we tested for the effect of name familiarity on whole-brain activation using voxelwise analyses of activation intensity for famous versus unfamiliar names in all participants. Figure 1 (top) displays the results from the tests of name familiarity effects on brain activation, which are detailed in Supplementary Table 2. Activation was greater for famous than unfamiliar names throughout the temporoparietal semantic network, frontal cortex, and bilateral caudate (not shown). Group differences in the effect of name familiarity (Figure 1, bottom) were found in the right cuneus and the left anterior middle temporal cortex, which were due to greater activation for famous than unfamiliar names in the control group [right cuneus: *F*(1,42) = 25.2, *p* < 0.00001, η_*p*_^2^ = 0.38; left anterior middle temporal: *F*(1,42) = 63.3, *p* < 6.5E-10, η_*p*_^2^ = 0.60], but not the PD group (*p* > 0.07).

#### Genetic Associations With Fame-Related Activation Intensity

Next, we tested whether each of the MAPT variants altered the effect of name familiarity on brain activation (Figure 1) in both groups. For these analyses, cortical ROI that showed greater activation for famous than unfamiliar names were extracted from the two large cluster volumes (Supplementary Table 2) to test for their associations with each of the MAPT polymorphisms. The ROI are described in Table 2. MANOVAs tested for the MAPT main effect and the group by MAPT interactions separately for four frontal, four parietal, four temporal, and two caudate (left and right) ROI. MAPT rs9468 and its interaction with group were not significantly associated with fame-related effects (famous > unfamiliar) for frontal, parietal or caudate ROI. There was a significant multivariate main effect of MAPT rs9463 on temporal cortex activation [*F*(4,99) = 3.1, *p* < 0.02, η_*p*_^2^ = 0.11]. Follow-up ANOVAs showed that this effect was localized to the right middle/inferior temporal cortex [*F*(1,102) = 5.1, *p* < 0.025, η_*p*_^2^ = 0.05] and at a subthreshold level, the right parahippocampus [*F*(1,102) = 3.9, *p* = 0.052, η_*p*_^2^ = 0.04], irrespective of group. These analyses showed that fame-related activation was greater in H1 homozygotes [right middle/inferior temporal: mean = 0.67 (0.05); right parahippocampus: mean = 0.42 (0.03)] than H2 carriers [right middle/inferior temporal: mean = 0.44 (0.09); right parahippocampus: mean = 0.30 (0.06)]. MAPT rs242557 and its interactions with group were non-significant for all ROI.

**TABLE 2:** Regions of interest showing greater famous than unfamiliar activation in voxelwise analyses.

Famous > Unfamiliar	*X, Y, Z*	Voxels
**Frontal**		
L medial/lateral superior/middle frontal, anterior cingulate	–7 57 22	8235
R medial/lateral superior/middle frontal, anterior cingulate	8 53 20	3516
L inferior frontal	–46 30 –3	2987
R inferior frontal	28 32 –14	586
**Parietal**		
L angular gyrus, inferior parietal	–49 –65 33	4832
R angular gyrus, inferior parietal	49 –67 32	2632
L precuneus, posterior cingulate	–7 –56 29	3117
R precuneus, posterior cingulate	7 –55 27	2209
**Temporal**		
L middle/inferior temporal	–58 –16 –17	6574
R middle/inferior temporal	52 5 –22	2692
L hippocampus, parahippocampus	–25 –23 –12	2053
R hippocampus, parahippocampus	26 –22 –14	1580
**Basal Ganglia**		
L caudate	–11 9 7	623
R caudate	11 11 7	611

*Regions of interest were extracted from two large cortical clusters (Supplementary Table 2) that showed greater activation for famous than unfamiliar names in voxelwise analyses.*

*X, Y, Z coordinates are based on the Montreal Neurological Institute (MNI) atlas.*

*L, left hemisphere; R, right hemisphere.*

### Group Differences in Fame-Modulated Functional Connectivity (gPPI)

The focus of the study was to test whether fame-modulated connectivity of regions that govern semantic processing differed between the PD and the control groups. To this end, the gPPI method first identified significant fame-modulated functional connections of a seed ROI with other brain regions in all subjects, and then tested for group differences in these connections. Supplementary Table 3 describes the coordinates for 19 seeds that were placed in cortical and caudate ROI where peak activation in the above voxelwise analyses was greater for famous than unfamiliar names or showed a group by familiarity interaction (Figure 1). All voxels within each 12 mm sphere were activated for each subject. Seven seeds were placed throughout lateral and medial frontal cortices, which broadly control access to semantic knowledge (Chiou et al., 2018). Five seeds were placed in parietal-occipital regions engaged by retrieval (e.g., precuneus, posterior cingulate) (Cavanna and Trimble, 2006; Ranganath and Ritchey, 2012; Jonker et al., 2018), semantic (inferior parietal, angular gyrus) (Binder and Desai, 2011; Price et al., 2015), and visual processes (cuneus). For temporal cortex, five seeds were placed in memory (parahippocampus) (Danker et al., 2017) and semantic (anterior middle temporal, inferior temporal) regions (Ralph et al., 2017). Two seeds were placed in the left and right caudate, which modulates semantic cognition in PD (Friederici, 2006; Canini et al., 2016).

**FIGURE 2:**
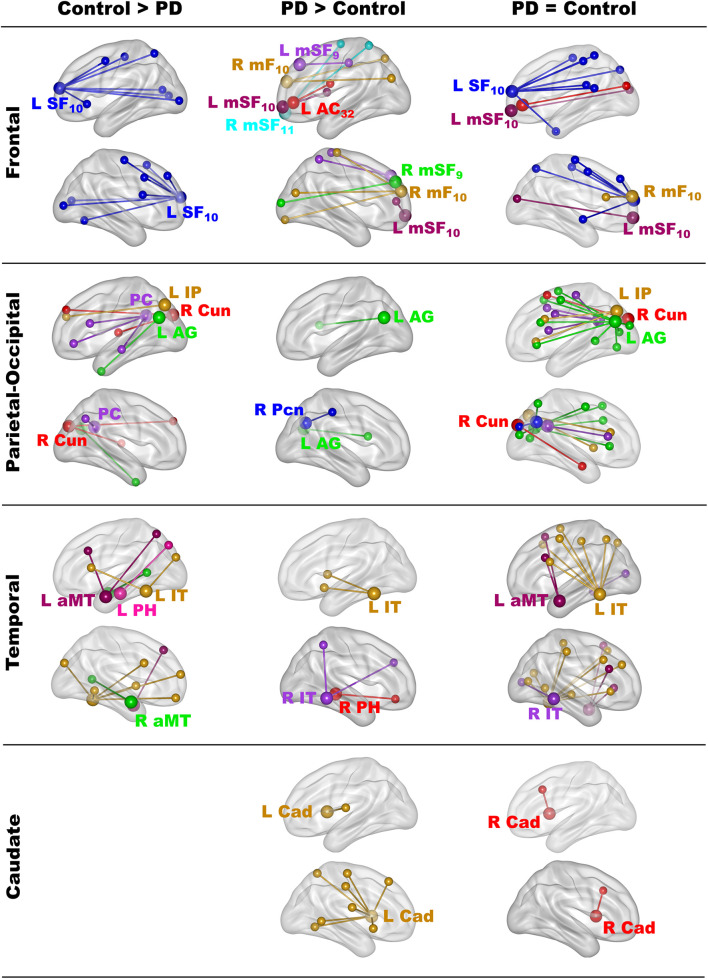
Group differences in fame-modulated functional connectivity. The figure illustrates the connections of a seed ROI (large balls) with other brain regions (small balls) that were stronger for famous than unfamiliar names. Seeds and their connections are color coded and separated into frontal, parietal-cuneus, temporal, and subcortical loci. In one or both groups, famous name seed time-courses correlated more positively with the time courses of other brain voxels than unfamiliar name seed time-courses. Columns display seed connections that were stronger in the control (Control > PD) or the PD group (PD > Control). The **right column** shows seed connections for which the strength of fame-modulated connectivity did not differ between groups (PD = Control). Supplementary Tables 4–6 detail the volumes and spatial coordinates of connectivity features, and the p values for tests of group differences in connectivity features. L, left hemisphere; R, right hemisphere; Brodmann areas are designated by subscripts. AC, anterior cingulate; AG, angular gyrus; aMT, anterior middle temporal; Cad, caudate; Cun, cuneus; IP, inferior parietal; IT, inferior temporal; mF, medial frontal; mSF, medial superior frontal; Pcn, precuneus; PC, posterior cingulate; PH, parahippocampus; SF, superior frontal.

The results showed that in one or both groups, famous name seed time-courses correlated more strongly (positively) with the time courses of other brain voxels than unfamiliar name seed time-courses. Figure 2 illustrates the 68 features that showed group differences in fame-modulated connectivity, which included 28 frontal, 13 parietal-occipital, 18 temporal and 9 left caudate features (for details see Supplementary Tables 4, 5). For some features, fame-related couplings (famous > unfamiliar names) were stronger in the control group than in the PD group (Figure 2, left column). For other features, fame-related couplings were stronger in the PD group than in the control group (Figure 2, middle column). In addition, 66 features showed fame-related couplings (famous > unfamiliar) that did not differ between the groups (Figure 2, right column and Supplementary Table 6), indicating preservation of these connections in PD.

### Principal Components Analysis of Abnormal Fame-Modulated Connectivity Features

**FIGURE 3:**
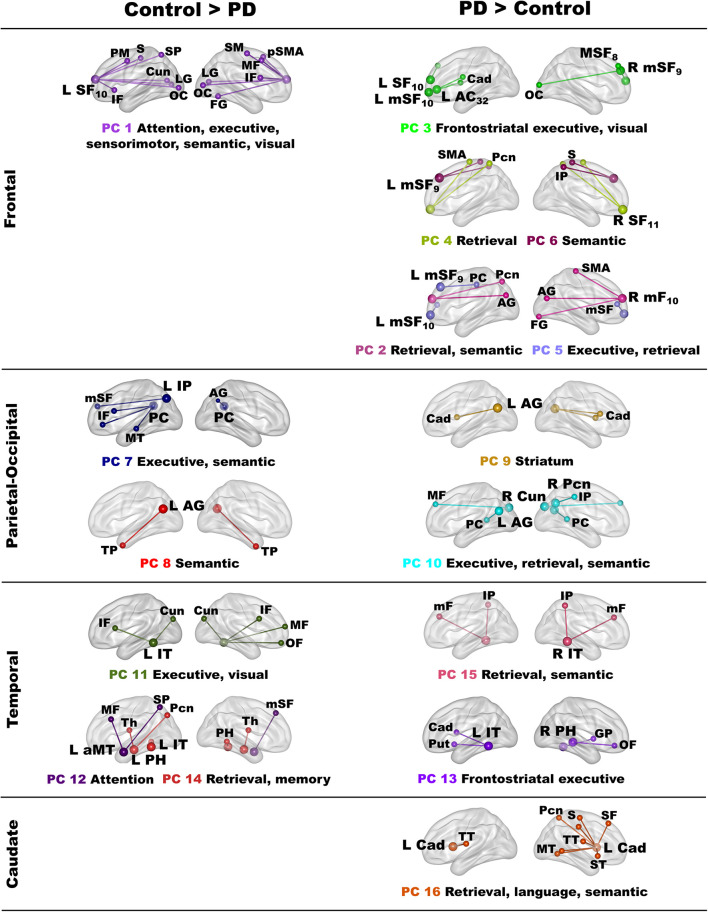
Principal components derived from abnormal fame-modulated connections. Separate principal component analyses were conducted for connectivity features associated with frontal, parietal-cuneus, temporal, and subcortical seeds. The **first and second columns** respectively illustrate the principal components (PC) for which scores were greater in the control than the PD group (control features) and greater in the PD than the control group (PD features). For each PC, the seed(s) and their connection(s) (small balls) are color coded. The connection topology of a seed(s) are briefly described by their connectivity with regions engaged by executive, attention, retrieval, memory, semantic and language processing systems. Sagittal sections display connectivity features for the left (L) and right (R) hemispheres. Tables 3, 4 detail the anatomy of features, matrix weights for each PC, and effect sizes from ANOVA tests for group differences in PC scores. Brodmann areas for frontal seeds are designated by subscripts. AC, anterior cingulate; AG, angular gyrus; aMT, anterior middle temporal; Cad, caudate; Cun, cuneus; FG, fusiform gyrus; IF, inferior frontal; GP, globus pallidus; IP, inferior parietal; IT, inferior temporal; LG, lingual gyrus; mF, medial frontal; mSF, medial superior frontal; MF, middle frontal; MT, middle temporal; OC, occipital cortex; OF, orbitofrontal; Pcn, precuneus; PC, posterior cingulate; PH, parahippocampus; PM, premotor; pSMA, presupplementary motor area; Put, putamen; S, sensory; SF, superior frontal; SM, sensorimotor; SMA, supplementary motor; SP, superior parietal; ST, superior temporal; Th, thalamus; TP, temporal pole; TT, transverse temporal.

Owing to the large number of fame-modulated connectivity features that differed between the groups (68 features), PCA was used to reduce them into components, rendering it more feasible for analyses of their associations with cognitive and genetic variables. PCA was performed separately for frontal, parietal-occipital, temporal, and left caudate seeds. PCs with eigenvalues ≥ 1.0 were extracted, resulting in six frontal, four parietal-occipital, and five temporal PCs, and one caudate PC (Tables 3, 4). Each feature loaded on a single component (i.e., weightings ≥ ± 0.41). Figure 3 illustrates the 16 components, which were characterized by the topology of a seed(s) connectivity with regions engaged by executive (frontal), attention (dorsal frontal, superior parietal), retrieval (SMA, precuneus, posterior cingulate), memory (parahippocampus), semantic (inferior parietal, middle/inferior temporal) and language processing (superior and transverse temporal) systems. PC scores reflect the strength of fame-modulated seed couplings with other brain regions. Positive PC couplings (famous > unfamiliar name connectivity) were features of the control or the PD group. Control features were positive PC couplings that were stronger in the control than in the PD group. PD features were positive PC couplings that were stronger in the PD than the control group.

**TABLE 3:** Principal components characterizing fame-modulated couplings of frontal and striatal regions of interest.

Hubs	Principal Components/Seeds	Connections	Weight^†^	η_*p*_^2^
**Frontal: executive Control > PD**	**PC 1: attention, executive, sensorimotor, semantic, and visual**			0.24
	L superior frontal (BA 10)	R middle frontal (BA 9,8)	0.82	
		L inferior frontal (BA 47)	0.76	
		R inferior frontal (BA 44)	0.66	
		R preSMA (BA 6)	0.81	
		L precentral (BA 6)	0.78	
		L superior parietal	0.68	
		R pre/postcentral (BA 3,4)	0.77	
		L postcentral (BA 3)	0.67	
		R fusiform (BA 37)	0.69	
		B lingual/cuneus (BA 18)	0.76	
		L cuneus (BA 18)	0.79	
		L middle/inferior occipital (BA 18)	0.73	
		R middle/inferior occipital (BA 18,19)	0.71	

**Frontal: executive PD > Control**	**PC 2: retrieval and semantic**			0.29
	R middle frontal (BA 10)	R SMA (BA 6)	0.59	
		L precuneus	0.84	
		L angular gyrus (BA 39)	0.77	
		R angular gyrus (BA 39)	0.72	
		R fusiform	0.70	
	**PC 3: frontostriatal executive and visual**			0.38
	L superior frontal (BA 10)	R middle frontal (BA 8)	–0.56	
	L middle superior frontal (BA 10)	L caudate body	0.62	
	L anterior cingulate (BA 32)	L caudate body	0.75	
	R medial superior frontal (BA 9)	R middle occipital (BA 18)	0.53	
	**PC 4: retrieval**			0.17
	R medial superior frontal (BA 11)	L SMA (BA 6)	0.85	
		L precuneus, superior parietal	0.85	
	**PC 5: executive and retrieval**			0.10
	L medial superior frontal (BA 10)	R medial superior frontal (BA 10)	0.66	
	L medial superior frontal (BA 9)	L posterior cingulate (BA 31)	0.48	
	**PC 6: semantic**			0.14
	L medial superior frontal (BA 9)	R paracentral (BA 5)	0.66	
		R inferior parietal	0.82	

**Caudate: cognitive control PD > Control**	**PC 16: retrieval, language and semantic**			0.31
	L caudate	R superior frontal (BA 8)	0.67	
		R precuneus	0.82	
		R postcentral (BA 2)	0.79	
		R postcentral (BA 3)	0.83	
		R postcentral (BA 3)	0.83	
		L transverse temporal (BA 42)	0.78	
		R transverse temporal (BA 42)	0.84	
		R superior temporal (BA 22)	0.77	
		R middle temporal (BA 21)	0.66	
		R middle temporal (BA 37)	0.77	

*^†^Values are pattern matrix weights except for PC 16, which are unrotated component matrix weights. Effect sizes from ANOVA tests for group differences in PC scores are designed by η_*p*_^2^.*

*L, left hemisphere; R, right hemisphere.*

**TABLE 4:** Principal components characterizing fame-modulated couplings of posterior cortical regions of interest.

Hubs	Principal Components/Seeds	Connections	Weight^†^	η_*p*_^2^
**Parietal: retrieval and semantic Control > PD**	**PC 7: executive and semantic**			0.42
	L inferior parietal (BA 40)	L medial superior frontal (BA 9)	0.75	
	Medial posterior cingulate (BA 31)	L inferior frontal (BA 47)	0.78	
		L inferior frontal (BA 45)	0.74	
		R angular gyrus (BA 39)	0.52	
		L middle temporal (BA 21)	0.67	
	**PC 8: semantic**			0.23
	L angular gyrus (BA 39)	L temporal pole (BA 36, 38)	0.91	
		R temporal pole (BA 36, 38)	0.90	

**Parietal: retrieval and semantic PD > Control**	**PC 9: striatum**			0.36
	L angular gyrus (BA 39)	B caudate body	0.82	
		R caudate body	0.81	
	**PC 10: executive, retrieval & semantic**			0.17
	R cuneus (BA 19)	L middle frontal (BA 10)	–0.41	
	R precuneus	R inferior parietal	0.72	
	L angular gyrus (BA 39)	B isthmus cingulate (BA 29)	0.60	

**Temporal: semantic and memory Control > PD**	**PC 11: executive and visual**			0.30
	L inferior temporal (BA 20)	R superior, middle frontal (BA 10)	0.81	
		R orbitofrontal (BA 11)	0.78	
		R inferior frontal (BA 9)	0.73	
		L inferior frontal (BA 44,45)	0.58	
		L cuneus (BA 19)	0.83	
		R cuneus (BA 19)	0.69	
	**PC 12: attention**			0.19
	L anterior middle temporal (BA 21)	L middle frontal (BA 9)	0.80	
		R medial superior frontal (BA 8)	0.69	
		L superior parietal (BA 7)	0.89	
	**PC 14: retrieval and memory**			0.31
	L PH	L precuneus	0.70	
		B thalamus/medial dorsal	0.83	
	L inferior temporal (BA 20)	R PH (BA 37)	0.60	

**Temporal: semantic and memory PD > Control**	**PC 13: frontostriatal executive**			0.37
	R PH	R orbitofrontal (BA 10)	0.60	
	L inferior temporal (BA 20)	L caudate body	0.61	
		L putamen	0.75	
		R GP	–0.52	
	**PC 15: retrieval and semantic**			0.18
	R inferior temporal (BA 20)	R medial frontal, preSMA (BA 9, 6)	0.80	
		R inferior parietal	0.79	

*^†^Values are pattern matrix weights. Effect sizes from ANOVA tests for group differences in PC scores are designed by η_*p*_^2^.*

*L, left hemisphere; R, right hemisphere; PH, parahippocampus.*

MANOVAs showed highly significant group differences for the six frontal [*F*(6,99) = 38.9, *p* < 0.001; η_*p*_^2^ = 0.70], four parietal-occipital [*F*(4,101) = 56.9, *p* < 0.001; η_*p*_^2^ = 0.69], and five temporal [*F*(5,100) = 50.5; *p* < 0.001; η_*p*_^2^ = 0.72] PC scores. Only one component was derived for the left caudate seed, which also showed robust group differences in PC scores [ANOVA; *F*(1,104) = 47.5, *p* < 0.001; η_*p*_^2^ = 0.31]. Follow-up ANOVAs showed that group differences in each PC score were associated with large effect sizes (Tables 3, 4; η_*p*_^2^ = 0.10 to 0.42). Disease duration, levodopa dosage equivalence, motor symptom severity (UPDRS Part 3), and symptoms of depression (Beck Depression Inventory) were not correlated with PC scores [FDR adjusted (*q* ≤ 0.05) separately for correlations of each clinical variable with 16 PC scores].

### PC Score Correlations With Semantic Cognition

**FIGURE 4:**
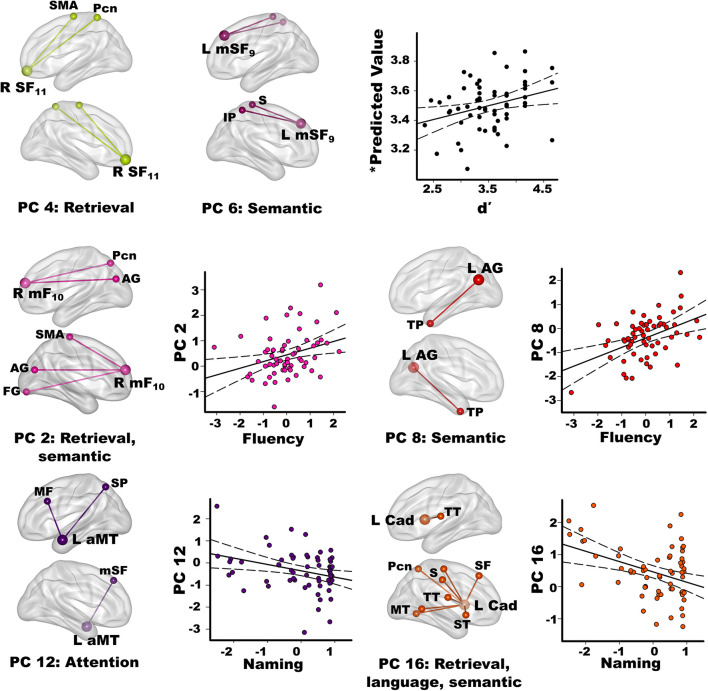
Relationships between semantic cognition and principal component scores in the PD group. Fame-discrimination (*d*’), category fluency (DKFES Category Fluency), and confrontation naming (Boston Naming Test) performances significantly correlated with specific principal component (PC) scores. For each PC, seed(s) (large balls) and their connection(s) (small balls) are color coded. Significant correlations between standardized PC scores (*y* axis) and measures of semantic cognition are shown on scatterplots for the PD group. Plots display the best-fitting linear regression line (solid line) and 95% conference intervals (dotted lines). Age adjusted residuals are plotted for Category Fluency and Confrontation Naming tests. For *d*’ **(top row)**, predicted values from the regression equation are plotted for frontal PC 4 and PC 6 [Σ intercept + (beta_*PC4*_ * PC 4 score) + (beta_*PC6*_ * PC 6 score) = Σ3.4 + (0.19 * PC 4 score) + (0.16 * PC 6 score)]. Brodmann areas for frontal seeds are designated by subscripts. L, left hemisphere; R, right hemisphere. AG, angular gyrus; aMT, anterior middle temporal; Cad, caudate; FG, fusiform gyrus; IP, inferior parietal; IT, inferior temporal; mF, medial frontal; mSF, medial superior frontal; MF, middle frontal; MT, middle temporal; Pcn, precuneus; PC, posterior cingulate; S, sensory; SF, superior frontal; SMA, supplementary motor; SP, superior parietal; ST, superior temporal; TP, temporal pole; TT, transverse temporal.

Stepwise multiple regressions were conducted separately for each group to test for sets of PC scores (frontal, parietal, temporal, caudate) that best explained individual differences in semantic cognition [FDR corrections (*q* ≤ 0.05) applied to uncorrected *p*-values; Figure 4]. For fame discrimination, higher *d*’ values in PD correlated with stronger PC 4 frontal couplings with retrieval circuits (*r*_*xy.z*_ = 0.29) and PC 6 frontal couplings with semantic regions (*r*_*xy.z*_ = 0.27) [*F*(2,59) = 4.7, *p* < 0.01, *q* < 0.05, *R* = 0.37], which were PD features. As both PCs positively correlated with *d*’, the predicted values from the regression equation are plotted in Figure 4, for which the correlation remained significant after removal of an outlier (*r* = 0.31, *p* < 0.015). In controls, *d*’ was not correlated with PC scores.

Better category fluency in PD correlated with stronger PC 2 frontal (PD feature) couplings with retrieval and semantic areas [*F*(1,61) = 6.7, *p* < 0.01, *q* < 0.04, *R* = 0.31] and stronger PC 8 parietal (control feature) couplings with a semantic-selective hub [*F*(1,61) = 12.5, *p* < 0.001, *q* < 0.01, *R* = 0.41]. Category fluency scores were not correlated with PC scores in the controls.

Better confrontation naming in PD correlated with weaker PC 12 temporal (control feature) couplings with attention areas [*F*(1,61) = 6.6, *p* < 0.01, *q* < 0.03, *R* = 0.31] and weaker PC 16 caudate (PD feature) couplings with retrieval, language processing, and semantic areas [*F*(1,61) = 10.9, *p* < 0.002, *q* < 0.02, *R* = 0.39]. Naming was not associated with PC scores in the controls.

### PC Score Correlations With Interfacing Cognitive Processes

**FIGURE 5:**
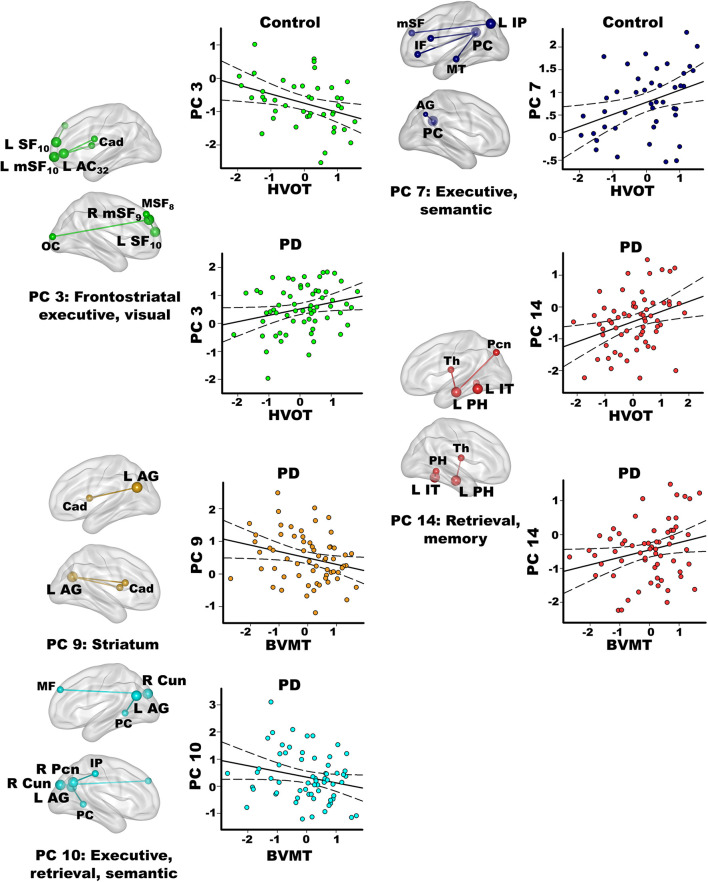
Relationships between principal component scores and interrelated cognitive functions. Visual organization and naming (Hooper Visual Organization; HVOT) and episodic memory (Brief Visuospatial Memory Test-Revised; BVMT) performances significantly correlated with specific principal component (PC) scores. For each PC, seed(s) (large balls) and their connection(s) (small balls) are color coded. Significant correlations between PC scores (*y* axis) and age adjusted residuals for the cognitive tests (*x* axis) are shown on scatterplots for the PD and the control groups. Plots display the best-fitting linear regression line (solid line) and 95% conference intervals (dotted lines). Brodmann areas for frontal seeds are designated by subscripts. L, left hemisphere; R, right hemisphere. AC, anterior cingulate; AG, angular gyrus; Cad, caudate; Cun, cuneus; IF, inferior frontal; IP, inferior parietal; IT, inferior temporal; mSF, medial superior frontal; MF, middle frontal; MT, middle temporal; OC, occipital cortex; Pcn, precuneus; PC, posterior cingulate; PH, parahippocampus; SF, superior frontal; Th, thalamus.

Stepwise multiple regressions were conducted separately for each group to test for test for sets of PC scores (frontal, parietal, temporal, caudate) that explained individual differences in processes that interface with semantic cognition (FDR adjusted; Figure 5). PC scores were not correlated with executive functions in either group (Inhibition/Switching, Letter Fluency).

In the PD group only, better visual memory (BVMT-R) correlated with weaker PC 9 parietal couplings with the caudate and weaker PC 10 parietal-cuneus couplings with executive, retrieval, and semantic regions [*F*(2,60) = 6.9, *p* < 0.005, *q* < 0.007, *R* = 0.41], which were PD features. Better visual memory in PD also correlated with stronger PC 14 temporal (control feature) couplings with retrieval and memory regions [*F*(1,61) = 4.2, *p* < 0.045, *p* = 0.05, *R* = 0.26]. In the control group, visual memory was not significantly correlated with PC scores. Verbal memory (CVLT-II) was not significantly related to PC scores in either group.

In the control group, better visual organization/naming performances (HVOT) correlated with weaker PC 3 frontal (PD feature) couplings with frontostriatal executive and visual areas [*F*(1,41) = 6.4, *p* < 0.016, *q* < 0.04, *R* = 0.37] and stronger PC 7 parietal (control feature) couplings with executive and semantic areas [*F*(1,41) = 6.5, *p* < 0.015, *q* < 0.03, *R* = 0.37]. In PD, better HVOT performances correlated with stronger PC 3 frontal (PD feature) couplings with frontostriatal executive and visual areas [*F*(1,61) = 5.5, *p* < 0.02, *q* < 0.028, *R* = 0.29] and stronger PC 14 temporal (control feature) couplings with retrieval and memory areas [*F*(1,61) = 8.2, *p* < 0.006, *q* < 0.01, *R* = 0.34].

### Genetic Associations With PC Scores

**FIGURE 6:**
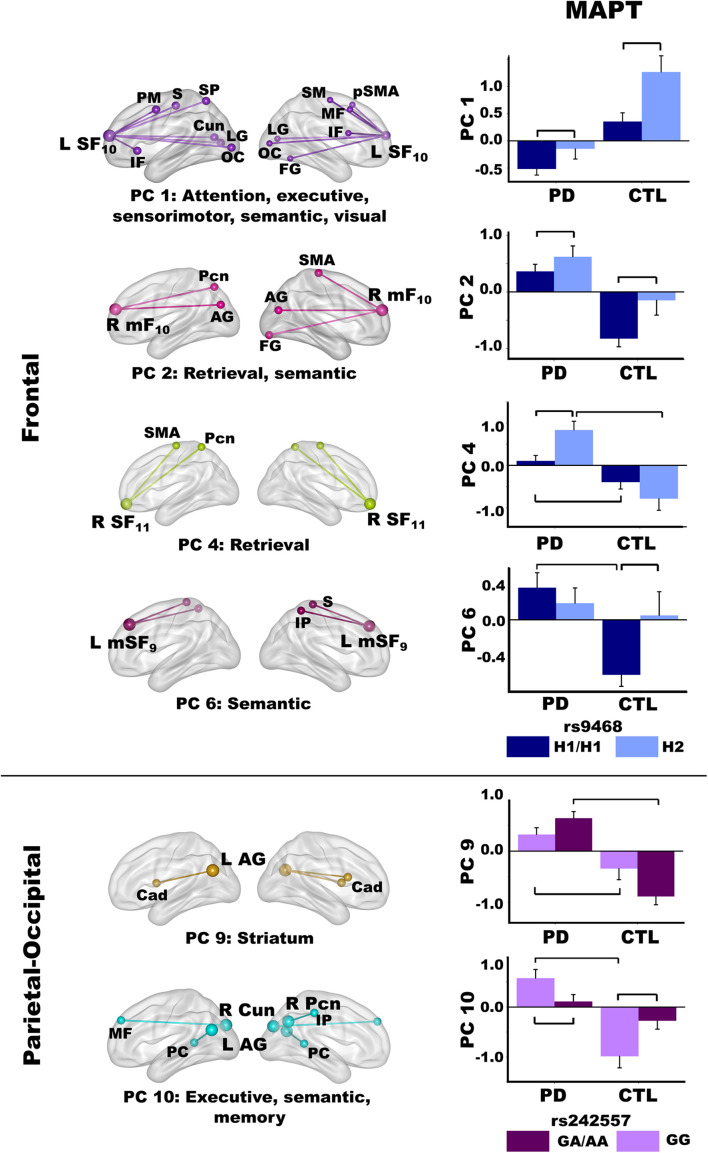
Relationships between MAPT and principal component scores. Significant effects of MAPT rs9468 and MAPT rs242557 were respectively observed for frontal and parietal-occipital principal components (PC). Means and standard error bars are plotted. Brackets above standard error bars designate the locus of significant MAPT effects. Brodmann areas for frontal seeds are designated by subscripts. L, left hemisphere; R, right hemisphere. AG, angular gyrus; Cad, caudate; Cun, cuneus; FG, fusiform gyrus; IF, inferior frontal; IP, inferior parietal; LG, lingual gyrus; mF, medial frontal; MF, middle frontal; OC, occipital cortex; Pcn, precuneus; PC, posterior cingulate; PM, premotor; pSMA, presupplementary motor area; S, sensory; SF, superior frontal; SM, sensorimotor; SMA, supplementary motor; SP, superior parietal.

Lastly, we tested whether MAPT variants altered the strength of PC coupling topologies. For each MAPT polymorphism, MANOVA models first tested for the main effect of MAPT and its interaction with group, separately for each set of PC scores (six frontal, four parietal, five temporal). Follow-up ANOVAs identified the source(s) of significant multivariate effects. For the caudate PC, ANOVA was used to test the main effect of MAPT and interactions with group, separately for each polymorphism. Figure 6 shows that MAPT rs9468 was only related to frontal PC scores and MAPT rs24557 was only related to parietal PC scores.

#### MAPT 9468

The MANOVA revealed a main effect of MAPT rs9463 for frontal PC scores [*F*(6,97) = 3.3, *p* < 0.005, η_*p*_^2^ = 0.17]. Follow-up ANOVAs showed that the effect was localized to PC 1 [*F*(1,102) = 12.1, *p* < 0.001, η_*p*_^2^ = 0.11] and PC 2 [*F*(1,102) = 6.6, *p* < 0.01, η_*p*_^2^ = 0.06]. In both groups, PC 1 (control feature) and PC 2 (PD feature) frontal couplings were more positive in H2 carriers than H1 homozygotes. The MANOVA also showed a group by rs9463 interaction [*F*(6,97) = 3.0, *p* < 0.01, η_*p*_^2^ = 0.15], which was localized to PC 4 [*F*(1,102) = 8.3, *p* < 0.005, η_*p*_^2^ = 0.08] and PC 6 [*F*(1,102) = 4.3, *p* < 0.04, η_*p*_^2^ = 0.04]. Here, PC 4 (PD feature) couplings were stronger in PD H2 carriers than PD H1 homozygotes [*F*(1,61) = 10.1, *p* < 0.002, η_*p*_^2^ = 0.14], whereas MAPT had no effect on PC 4 connectivity in controls. In addition, group differences in PC 4 scores were striking for H2 carriers [*F*(1,29) = 22.7, *p* < 0.0001, η_*p*_^2^ = 0.44], but still significant for H1 homozygotes [*F*(1,73) = 6.2, *p* < 0.015, η_*p*_^2^ = 0.08]. In contrast, PC 6 (PD feature) couplings were more negative in control H1 homozygotes than H2 carriers [*F*(1,41) = 6.1, *p* < 0.018, η_*p*_^2^ = 0.13], but MAPT was not related to PC 6 connectivity in the PD group. Moreover, group differences in PC 6 scores were found for H1 homozygotes [*F*(1,73) = 19.1, *p* < 0.0001, η_*p*_^2^ = 0.21], but not H2 carriers.

#### MAPT rs242557

For the MAPT rs242557 polymorphism, the MANOVA showed a group by MAPT interaction for parietal PC scores [*F*(4,99) = 4.0, *p* < 0.005, η_*p*_^2^ = 0.14]. Follow-up ANOVAs showed the interaction effect was localized to PC 9 [*F*(1,102) = 7.0, *p* < 0.008, η_*p*_^2^ = 0.07] and PC 10 [*F*(1,102) = 10.2, *p* < 0.002, η_*p*_^2^ = 0.09]. In both groups, there was a subthreshold trend (medium effect sizes) for PC 9 (PD feature) couplings to be stronger in people with higher (A) than lower (GG) tau activity [PD: *F*(1,61) = 3.0, *p* = 0.08, η_*p*_^2^ = 0.05; Control: *F*(1,41) = 3.7, *p* = 0.06, η_*p*_^2^ = 0.08]. In higher tau activity carriers (A), PC 9 connectivity was also markedly strengthened in the PD group relative to controls [*F*(1,61) = 56.1, *p* < 0.0001, η_*p*_^2^ = 0.48], whereas group differences were not as large in GG carriers with lower tau activity [*F*(1,41) = 7.3, *p* < 0.01, η_*p*_^2^ = 0.15]. In contrast, PC 10 (PD feature) couplings were stronger in people with lower (GG) than higher (A) tau activity [PD: *F*(1,61) = 4.5, *p* < 0.038, η_*p*_^2^ = 0.07; Control: *F*(1,41) = 5.7, *p* < 0.02, η_*p*_^2^ = 0.12]. Group differences in PC 10 couplings were found only for GG carriers [*F*(1,41) = 26.3, *p* < 0.001, η_*p*_^2^ = 0.39].

### Genetic Associations With Cognitive Variables

Multivariate analyses of variances tests for the main effect of each MAPT polymorphism and interactions with group were non-significant for all semantic measures (*p* > 0.57) and other cognitive variables (*p* > 0.20).

## Discussion

Many studies of PD report semantic fluency and naming impairments in the absence of MCI, as well as deficient processing of affective and action-related semantic content (Auclair-Ouellet et al., 2017). However, scant attention has been paid to the underlying neurophysiopathological mechanisms. This study demonstrated, for the first time, marked changes in the connectivity of the brain during recollection of semantic content unrelated to actions (Bocanegra et al., 2015) in cognitively normal PD. Proper names is a semantic category that is often vulnerable in normal aging, wherein retrieval difficulties are associated with gray matter loss in frontal and inferior parietal, but not temporal cortex (Kljajevic and Erramuzpe, 2018). In the present study, nearly half of the fame-modulated frontal, parietal, temporal, and right caudate connections were preserved in PD, which may help sustain semantic processing. However, two aberrant connectivity patterns were uncovered in PD, which were reduced into principal components that reflected the strength of fame-modulated regional couplings with other brain areas. One pattern was related to a loss in frontal, parietal, and temporal connection topologies that governed semantic recollection in the control group (control features). Another pattern was characterized by functional reconfiguration, wherein frontal, parietal, temporal and left caudate couplings were strengthened with areas not recruited by the control group (PD features). The functional significance of component topologies was demonstrated by their correlations with semantic cognition and interrelated processes, which distinguished PC circuits by their compensatory and detrimental influences on different facets of cognition. Moreover, MAPT risk variants adversely altered the strength of frontal and parietal coupling topologies, sometimes in both PD and healthy controls. Increased tau transcription diminished recruitment of compensatory circuits and amplified recruitment of connection topologies that may adversely influence some cognitive functions. MAPT did not correlate with cognitive measures, possibly signifying that in cognitively normal adults genetic traits are a more intermediate phenotype of neurological processes than cognitive proficiency (Meyer-Lindenberg and Weinberger, 2006).

### Control and PD Connection Topologies

In the control group, the functional architecture of regional coupling topologies during semantic recollection broadly aligned with models that assume the process of remembering is multifaceted (Binder and Desai, 2011; Ralph et al., 2017). Stronger expression of certain parietal and temporal control topologies (PC 7, PC8, PC 14) also correlated with better cognition in both groups (see section “Associations Between Component Topologies and Semantic Cognition”), bolstering their normal role in semantic cognition. Frontal components characterized connections of regions that control access to semantic content. In the control group, left superior-frontal (PC 1) couplings were strengthened with regions involved in attention (superior frontal, superior parietal), executive (inferior/middle frontal), sensory, semantic (fusiform) and visual processing. This contrasted with the PD group wherein fame recollection was associated with strengthened connectivity of multiple frontal regions (PC 2 – PC 6), suggesting that access to semantic content depended upon recruitment of diverse frontal circuits. Frontal component topologies also differed from controls by their strengthened connections with retrieval areas (SMA, precuneus, posterior cingulate) (Fairhall and Caramazza, 2013) and recruitment of multiple semantic hubs (angular gyrus, inferior parietal, fusiform gyrus) (Binder and Desai, 2011; Price et al., 2015), demonstrating a migration of connectivity to regions that were not recruited by controls. These results may signify greater involvement of semantic control processes, perhaps owing to the difficulty of accessing semantic details associated with famous names.

In contrast, parietal-occipital components in controls reflected the connectivity of regions normally engaged by retrieval (Cavanna and Trimble, 2006; Ranganath and Ritchey, 2012; Jonker et al., 2018), semantic (Price et al., 2015), and visual systems. Inferior parietal and posterior cingulate (PC 7) couplings were strengthened with executive regions, including the left inferior frontal gyrus, which governs semantic selection (Canini et al., 2016; Cousins and Grossman, 2017; Xu et al., 2020), and semantic hubs. Couplings of the left angular gyrus were strengthened with the bilateral temporal poles (PC 8), for which atrophy is striking in semantic dementia and the semantic variant of primary progressive aphasia (Gorno-Tempini et al., 2011; Ralph et al., 2017). Thus, the control group engaged multiple semantic hubs during fame recollection possibly due the greater difficulty or depth of processing semantic content for famous names. The PD group failed to amplify these connection topologies, perhaps signifying impoverished representations of semantic content. Instead, the PD group showed strengthened precuneus and angular gyrus (PC 9, 10) couplings with alternative regions not recruited by the controls, indicating functional reconfiguration of semantic connections.

Temporal components in controls reflected coupling topologies of semantic (inferior/anterior middle temporal) and memory (parahippocampus) hubs (Jackson et al., 2016). Fame recollection strengthened left inferior temporal couplings (PC 11) with frontal cortex and the cuneus, for which activation intensity was also increased for famous names in controls, possibly reflecting reactivation of visual images of famous people (Danker and Anderson, 2010). Left anterior middle temporal couplings (PC 12) were strengthened with frontoparietal dorsal attention areas (Braga et al., 2013). PC 14 couplings reflected the normal interface between semantic and memory hubs, which support semantic recollection (Ranganath and Ritchey, 2012). These coupling topologies were lost in the PD group and replaced by strengthened left inferior temporal cortex (PC 13) couplings with different areas. Strengthened right inferior temporal (PC 13) and right parahippocampus (PC 15) coupling topologies also emerged, signifying recruitment of bilateral temporal cortices during fame recollection in PD to retrieve semantic content, such as visual details about famous people.

In PD fame recollection was also characterized by strengthened connectivity of the left caudate (PC 16) with retrieval, semantic memory, and language processing (superior temporal, Heschl’s gyrus, somatosensory) areas. Though the caudate’s role in semantic cognition is not well understood, in PD caudate communications with cortex are thought to reflect engagement of conscious control processes (Friederici, 2006).

### Associations Between Component Topologies and Semantic Cognition

Connection topologies were associated with different measures of semantic memory, some of which predict PD MCI and dementia. The results were not related to executive functioning, which did not correlate with PC scores. In PD, better fame discrimination correlated with stronger medial superior frontal couplings with retrieval (PC 4) and semantic hubs (PC 6) (PD features), which aligns with the role of medial frontal cortices in accessing people-related information (Fairhall and Caramazza, 2013). The results also suggest that recruitment of frontal couplings with retrieval and semantic (inferior parietal) areas may support compensatory routes for recollection.

Different connection topologies correlated with category fluency, a risk factor for later dementia in PD (Williams-Gray et al., 2013). Category fluency, but also phonemic fluency tests are widely used to assess executive and language dysfunction. Phonemic fluency enlists phonological search strategies for words constrained by their first letter, whereas category fluency enlists semantic search processes for words that belong to specific categories. These differences likely explain why semantic recollection topologies failed to correlate with letter fluency in our study. Poorer semantic but not phonemic fluency correlates with frontal and parietal-occipital thinning in PD (Pereira et al., 2014), which is compatible with our results. Specifically, stronger right medial frontal (PC 2; PD feature) connectivity with retrieval (Fairhall and Caramazza, 2013) and semantic areas (Price et al., 2015) correlated with better category fluency, suggesting that functional reconfiguration supported a compensatory route for semantic access. Better category fluency in PD also correlated with strengthened left angular gyrus (PC 8; control feature) couplings with the semantic-selective temporal poles (Ralph et al., 2017), reinforcing this circuit’s normal role in semantic cognition. Longitudinal studies are needed to determine if connectivity changes in these circuits may be markers of semantic memory decline.

Word finding is another facet of semantic memory that is tested by confrontation naming, which requires generating names of pictures. Naming distinguishes cognitively normal PD from PD MCI (Biundo et al., 2014), and predicts conversion to dementia (Hobson and Meara, 2015). In healthy people naming activates middle temporal semantic areas (Abrahams et al., 2003; Moriai-Izawa et al., 2012), which is compatible with our finding that naming in PD was related to the strength of left anterior middle temporal couplings (PC 12; control feature) with dorsal attention areas (Braga et al., 2013). Yet stronger PC 12 couplings were correlated with poorer naming in PD. Outwardly this finding is surprising since communications between semantic hubs and top-down attention systems might be expected to improve semantic access. However, PC 12 scores did not correlate with d’ in either group, possibly due to the low attention demands of the task. For the Boston Naming test, however, common object names are automatically activated whereas finding uncommon object names requires attentional control (Hoffman et al., 2015). Patients with poorer naming may therefore engage attention to find names owing to failed automatic reactivation of semantic details, which may be a sign of impoverished representations of semantic content. This hypothesis is compatible with our finding that poorer naming in PD was also related to strengthened caudate couplings (PC 16; PD feature) with a semantic hub (middle temporal) and phonological processing centers that support language. The caudate plays a supervisory role in language selection and semantic cognition (Abutalebi et al., 2008; Canini et al., 2016), and is activated when language processes cannot rely on automatic mechanisms (Friederici, 2006). Thus, strengthened caudate couplings with language areas, as well as retrieval and semantic circuits, may reflect engagement of covert verbal strategic searches to access names that cannot be automatically retrieved.

### Component Topology Relationships With Interfacing Cognitive Functions

Semantic recollection circuitry also showed unique associations with interdependent cognitive functions including visual episodic memory (BVMT). Strengthened left parahippocampus and inferior temporal couplings (PC 14; control feature) with retrieval and memory areas was beneficial for delayed recall in PD, in further support of this circuit’s normal role in semantic recollection. In contrast, poorer memory correlated with stronger recruitment of parietal circuits (PC 9, PC 10) that were features of PD. In this regard, the angular gyrus integrates visuospatial details of events to build coherent representations (Price et al., 2015). Stronger parietal connections with regions not recruited by the control group may therefore signify impoverished representations of visual content, perhaps owing declining visuospatial cognition, which was found in our PD group.

Coupling topologies also correlated with the HVOT, which tests naming of object drawings that are dismantled into puzzle-like pieces. The HVOT primarily measures perceptual organization abilities, but memory, executive functions, and confrontation naming are component processes (Merten, 2005; Jefferson et al., 2006; Higginson et al., 2011). Indeed, better HVOT performances in PD also correlated with stronger inferior temporal couplings (PC 14) with retrieval and memory regions, suggesting this circuitry facilitates semantic recollection. Interestingly, stronger frontal couplings with bilateral caudate and visual areas (PC 3; PD feature) also correlated with better HVOT performances in PD, perhaps reflecting beneficial effects of frontostriatal modulation to organize picture fragments into nameable objects. However, control participants who expressed this PD feature showed poorer HVOT performances, signifying adverse effects of recruiting this abnormal connection topology. Rather, in controls better HVOT performances correlated with stronger couplings of parietal regions (PC 7; control feature) with semantic hubs (middle temporal, angular gyrus) and frontal executive areas, in alignment with the naming and executive components of the test.

### Genotypes

To our knowledge this is the first investigation into the relationships between MAPT and functional connectivity during semantic recollection. Although tau is expressed throughout the brain (Trabzuni et al., 2012; Rittman et al., 2016), regional vulnerabilities to MAPT risk variants are not understood in PD. In pathologically confirmed PD cases, H1 homozygotes had higher overall neocortical and parietal cortex Lewy Body counts relative to H2 carriers (Robakis et al., 2016). This partly aligns with greater frontal and posterior cortical volume loss in de novo PD H1 homozygotes, which predicted poorer cognitive outcome (Sampedro et al., 2018). Our study extends these findings, demonstrating the vulnerability of frontal and parietal connectivity topologies to increased tau transcription. Medium to very large effect sizes characterized these relationships, suggesting that MAPT polymorphisms may be useful in explaining heterogeneous changes in brain functioning underlying semantic memory.

Regardless of disease status, H1 homozygotes exhibited weakened connectivity of two frontal regions. PC 1 couplings (control feature) were markedly weaker in control H1 homozygotes than control H2 carriers, whereas PD H1 homozygotes showed decidedly negative fame-modulated connectivity. These results are compatible with frontal and temporal gray matter loss in healthy adult H1 homozygotes (Canu et al., 2009), possibly reflecting emergent pathology. The reverse pattern was observed for PC 2 couplings (PD feature), whose connectivity strength correlated with better category fluency in PD. PC 2 couplings were weaker in PD H1 homozygotes than PD H2 carriers, suggesting increased tau transcription may diminish recruitment of compensatory circuitry. This finding may be related to Lewy body pathology associated with the H1 haplotype (Colom-Cadena et al., 2013b; Heckman et al., 2019). In control H2 carriers, PC 2 couplings were absent, suggesting a protective effect of the H2 haplotype (Colom-Cadena et al., 2013b; Zhang et al., 2017) against recruitment of PD features. Group differences in MAPT expression were also found for frontal PC 4 and PC 6 (PD features), whose connectivity strengths correlated with better fame discrimination in PD. Right medial superior frontal couplings (PC 4) were nearly absent in PD H1 homozygotes, demonstrating impoverished recruitment of compensatory circuitry. In healthy controls, however, the H2 haplotype protected against recruitment of non-normative left medial superior frontal couplings (PC 6), whereas H1 homozygotes showed markedly negative couplings. Notably, MAPT expression did not alter frontal cortex activation intensity, consistent with another study (Nombela et al., 2014).

In contrast, tau H1 transcriptions levels altered the strength of parietal-occipital couplings (PD feature), for which stronger connectivity correlated with poorer visual memory in PD. In both groups, angular gyrus couplings with the caudate (PC 9) tended to be stronger in higher tau activity A carriers than lower tau GA/AA carriers (medium effect sizes). However, group differences in connectivity strength were striking for A carriers (very large effect sizes), indicating that the A allele amplified the effect of the disease. Correspondingly, PD H1 homozygotes who were not screened for MCI showed hypoactivation of parietal cortex and the caudate during a spatial rotation task (Nombela et al., 2014). Collectively, these findings are compatible with the greater Lewy body pathology in parietal cortex in H1 homozygotes (Robakis et al., 2016), which adversely alter both parietal activation and connectivity. Group differences in the strength of parietal-cuneus couplings (PC 10), however, were specific to lower tau transcription PD and control GG carriers, suggesting pathological mechanisms of the disease likely explain recruitment of this circuit.

Microtubule-associated protein tau did not influence connectivity strengths of temporal regions. Rather, in both groups activation intensity (famous > unfamiliar) of the right middle/inferior temporal cortex and parahippocampus was greater in H1 homozygotes than H2 carriers, perhaps suggesting compensation for emerging or accelerated pathology (Reuter-Lorenz and Park, 2014; Gomperts et al., 2016; Scholl et al., 2016; Shen et al., 2019). Indeed, cognitively intact elders at genetic risk for Alzheimer’s disease showed hyperactivity of middle temporal and hippocampal regions during fame discrimination, which declined over 5 years in concert with episodic memory decline (Rao et al., 2015). Thus, compensation may be diminished or exhausted as neuropathology accumulates and cognitive deficits emerge. This aligns with findings in PD and control cohorts who were not screened for MCI, wherein H1 homozygotes exhibited hypoactivation of the parahippocampus and inferior temporal cortex and poorer episodic memory (Winder-Rhodes et al., 2015).

### Limitations

Several limitations should be considered, including that patients were tested on medication, which could mask functional abnormalities. In this regard, drug naïve patients are a more ideal group to study. Nonetheless, abnormal functional connectivity topologies were robust, likely owing in part to the improved temporal resolution of our multiband fMRI protocol (Tomasi et al., 2016). From a practical standpoint, it is also important to understand brain functioning in daily life as influenced by patients’ medication therapy. Second, neurocognitive correlations were typically medium in magnitude, likely owing to the more restricted ranges on behavioral variables in cognitively normal PD cohorts relative to studies of mixed PD cohorts with and without MCI. Compensatory processes in cognitively normal PD could also improve cognition and mask cognitive difficulties (Reuter-Lorenz and Park, 2014), thereby minimizing neurocognitive associations. The fidelity or coherence of regional connectivity may also be reduced for PD coupling topologies, which should increase variability such that connectivity might not correlate well or possibly at all with performance. Third, our PD sample was large, but the statistical power of genetic tests would be greater with larger samples. Even so, MAPT effects were associated with medium to large effect sizes and consistent with a study of a small PD cohort (*n* = 37) (Winder-Rhodes et al., 2015). Fourth, most but not all PC couplings topologies were derived from left hemisphere regions, owing to the left hemisphere bias for processing verbal rather than non-verbal materials, which are biased for right hemisphere processing (Rice et al., 2015; Ralph et al., 2017). Still, verbal and pictorial stimuli activate semantic systems, and performances on famous name and famous face discrimination tests are both impaired in semantic dementia (Snowden et al., 2004). Lastly, consideration of potential sex differences in semantic cognition fell outside the scope of the present study. However, this is an important avenue for research that stands to unravel mechanisms for heterogeneity in semantic decline in PD as emerging research suggests that semantic processing of specific categories (action fluency) may be sex linked in PD and related to frontal-temporal activation (Auclair-Ouellet et al., 2021).

## Conclusion

The results provide a new understanding of early vulnerabilities in the functional architecture of regional connectivity in PD during semantic recollection. Abnormal connectivity patterns were partly related to a loss in frontal, parietal, and temporal connection topologies that govern semantic cognition and interrelated processes in healthy elders. PD patients also showed functional reconfiguration, which was characterized by the migration of connectivity to areas that were not recruited by fame recollection in controls. Reconfiguration of frontal circuits supported compensatory routes for accessing semantic content in PD, which aligns with the prominence of frontal circuitry in compensatory processes in older adults (Reuter-Lorenz and Park, 2014). In contrast, reconfigured parietal and temporal connection topologies were detrimental or unrelated to cognition. The results also suggested that stronger enlistment of caudate circuitry for semantic recollection in PD may be a sign of weakened automatic reactivation of semantic content, possibly owing to impoverished semantic representations. In addition, strengthened recruitment of control features, namely parietal and temporal couplings with semantic and memory retrieval areas (PC 8, PC 14), was related to better cognition in PD underscoring individual differences in the preservation of semantic network circuitry. Our results build upon past research (Winder-Rhodes et al., 2015), showing that increased tau transcription alters the intensity of temporal cortex activation during fame recollection in both PD and healthy aging. We further demonstrated that increased tau transcription diminished recruitment of normal frontal connection topologies in healthy older adults, whereas it reduced or prevented recruitment of compensatory circuitry in PD and promoted recruitment of a parietal circuit that was adversely related to cognition. These preliminary findings indicate that tau transcription may explain some individual differences brain functioning and cognition in PD and normal aging. Longitudinal imaging genetics studies are needed to identify functional topologies that track cognitive progression and predict future cognitive status. Outcomes from this research could inform strategies for selecting patients for clinical trials based on their genetic profiles and have implications for identifying people who stand to benefit from therapeutic interventions that depend on the capacity to recruit compensatory circuitries that maintain cognition.

## Data Availability Statement

The data that support the findings of this study are available on reasonable request from the corresponding author. The data are not publicly available due to privacy or ethical restrictions imposed by the U.S. Department of Veterans Affairs. Genetic variant data from this study are deposited in dbSNP (https://www.ncbi.nlm.nih.gov/SNP/snp_viewTable.cgi?handle=COGNITIONPD).

## Ethics Statement

The studies involving human participants were reviewed and approved by Institutional Review Board, VA San Diego Healthcare System. The patients/participants provided their written informed consent to participate in this study.

## Author Contributions

DH conceived and designed the study, performed the statistical analyses, and wrote the first draft of the manuscript. QS acquired the data and performed the neuroimaging analyses. VS and XW acquired the data and organized the databases. RL reviewed the brain MRIs. MH, IL, QS, and VS contributed to the manuscript revision. All the authors read and approved the submitted version.

## Conflict of Interest

The authors declare that the research was conducted in the absence of any commercial or financial relationships that could be construed as a potential conflict of interest.

## Publisher’s Note

All claims expressed in this article are solely those of the authors and do not necessarily represent those of their affiliated organizations, or those of the publisher, the editors and the reviewers. Any product that may be evaluated in this article, or claim that may be made by its manufacturer, is not guaranteed or endorsed by the publisher.

## Footnotes

1
https://www.dnagenotek.com

2
http://afni.nimh.nih.gov

3
http://www.fmrib.ox.ac.uk/fsl/
