# Supplementary material for: Semantic Recollection in Parkinson’s Disease: Functional Reconfiguration and MAPT Variants
Source: Front Aging Neurosci. 2021 Sep 20;13:727057. doi: 10.3389/fnagi.2021.727057 (PMC8489380; doi:10.3389/fnagi.2021.727057)
Supplement: Supplementary file 1 [file Table_1.docx]

**Supplementary Table 1. Allele frequencies for MAPT genes.**

|  |  | **MAPT rs24557** | |
| --- | --- | --- | --- |
| **MAPT rs9468** | **Parkinson’s Group** | ***GG*** | **AG/AA** |
|  | **H1/H1** | 15 | 28 |
|  | **H2** | 15 | 5 |
|  | **Control Group** |  |  |
|  | **H1/H1** | 7 | 25 |
|  | **H2** | 6 | 5 |
