# Supplementary material for: Semantic Recollection in Parkinson’s Disease: Functional Reconfiguration and MAPT Variants
Source: Front Aging Neurosci. 2021 Sep 20;13:727057. doi: 10.3389/fnagi.2021.727057 (PMC8489380; doi:10.3389/fnagi.2021.727057)
Supplement: Supplementary file 2 [file Table_2.docx]

**Supplementary Table 2. Significant effects of fame discrimination on regional activation**

| **Condition Main Effect** | | | |
| --- | --- | --- | --- |
| **Famous > Unfamiliar** | **Brodmann Areas** | **X, Y, Z** | **Voxels** |
| B SF, mSF, MF, mF, AC, PC, Cad, PH, ST, Amygdala L inferior frontal, Pcn, angular gyrus, IP | 8,9,10,11,21,47,31,32  7,47, 39, 40 | -21 2 18 | 53332 |
| R MT, ST, IT, Pcn, IP, superior occipital | 20,21,22,7,40,19 | 51 -62 26 | 4068 |
| R inferior semi-lunar lobule, tonsil, pyramis |  | 32 -72 -40 | 2968 |
| B tonsil |  | 3 -53 -47 | 692 |
| L inferior semi-lunar lobule |  | -38 -72 -39 | 160 |
| L inferior semi-lunar lobule |  | -15 -83 -41 | 70 |
| **Unfamiliar > Famous** |  |  |  |
| L precentral, postcentral, IP | 6,4,2,3,40 | -48 -18 53 | 4300 |
| L middle/inferior occipital | 18,19 | -35 -94 4 | 1242 |
| R inferior frontal, precentral | 44,9 | 59 9 23 | 353 |
| R SF, MF | 9,10 | 39 52 28 | 344 |
| L SMA | 6 | -5 -1 60 | 247 |
| R inferior semi-lunar lobule |  | 18 -67 -54 | 217 |
| R preSMA, mF, AC | 8,6,32 | 6 26 44 | 187 |
| L cuneus | 19 | -50 -66 -6 | 110 |
| R inferior occipital | 18 | 34 -96 -5 | 69 |
| R precentral | 6 | 38 -7 72 | 59 |
| R insula | 13 | 40 20 3 | 40 |
| L Pcn | 7 | -22 -66 53 | 40 |
| R declive |  | 25 -63 -22 | 20 |
| L ST, insula | 13,42 | -54 -31 20 | 19 |
| **Group by Condition Interaction** | | | |
| **Famous > Unfamiliar** |  |  |  |
| R cuneus | 18,19 | 5 -93 24 | 250 |
| L MT | 38 | -63 4 -23 | 187 |

X, Y, Z coordinates are based on the Montreal Neurological Institute (MNI) atlas.

Bilateral=B; L=left hemisphere; R=right hemisphere. AC = anterior cingulate; IP = inferior parietal; IT = inferior temporal; mF = medial frontal; mSF = medial superior frontal; MF = middle frontal; MT = middle temporal; preSMA = pre-supplementary motor area; SF = superior frontal; Pcn = precuneus; PC = posterior cingulate; PH = parahippocampus/hippocampus; SF = superior frontal; ST = superior temporal; SMA = supplementary motor area
