# Supplementary material for: Semantic Recollection in Parkinson’s Disease: Functional Reconfiguration and MAPT Variants
Source: Front Aging Neurosci. 2021 Sep 20;13:727057. doi: 10.3389/fnagi.2021.727057 (PMC8489380; doi:10.3389/fnagi.2021.727057)
Supplement: Supplementary file 3 [file Table_3.docx]

**Supplementary Table 3. Seed coordinates for gPPI functional connectivity analyses**

| **Seed** | **MNI Coordinates** |
| --- | --- |
| **Frontal** |  |
| R medial superior (mSF, BA 11) | 17 55 -13 |
| L superior frontal (SF, BA 10) | -11 74 17 |
| L medial superior frontal (mSF, BA 9) | -10 51 41 |
| R medial superior frontal (mSF, BA 9) | 10 51 41 |
| R medial frontal (mF, BA 10) | 7 58 23 |
| L medial superior frontal (mSF, BA 10) | -6 61 -6 |
| L anterior cingulate (AC, BA 32) | -6 45 -1 |
| **Parietal-Occipital** |  |
| medial posterior cingulate (mPC, BA 31) | 0 -50 28 |
| R precuneus (Pcn, BA 7) | 6 -64 33 |
| L inferior parietal (IP, BA 40) | -47 -73 41 |
| L angular gyrus (AG, BA 39) | -52 -66 26 |
| R cuneus (BA 19) | 5 -91 31 |
| **Temporal** |  |
| L anterior middle temporal (aMT, BA 21) | -57 0 -21 |
| L inferior temporal (IT, BA 20) | -61 -50 -16 |
| R inferior temporal (IT, BA 20) | 62 -44 -10 |
| L parahippocampus (PH) | -29 -18 -19 |
| R parahippocampus (PH) | 29 -33 -5 |
| **Subcortical** |  |
| L caudate | -10 11 7 |
| R caudate | 10 11 7 |

Brodmann area=BA; Montreal Neurological Institute (MNI) brain atlas coordinates.
