# Supplementary material for: Semantic Recollection in Parkinson’s Disease: Functional Reconfiguration and MAPT Variants
Source: Front Aging Neurosci. 2021 Sep 20;13:727057. doi: 10.3389/fnagi.2021.727057 (PMC8489380; doi:10.3389/fnagi.2021.727057)
Supplement: Supplementary file 4 [file Table_4.docx]

**Supplementary Table 4.** Fame-modulated couplings that were stronger in the control than in the PD group.

| **Seed** | **Region (Brodmann area)** | **Voxels** | **MNI^†^** | **p value^‡^** |
| --- | --- | --- | --- | --- |
| **Frontal** | **Control > PD** |  |  |  |
| L SF (BA 10) | R middle frontal (BA 8,9) | 306 | 54 13 40 | 2.00E-06 |
|  | R middle frontal (BA 8) | 123 | 38 43 44 | 1.50E-05 |
|  | R inferior frontal (BA 44) | 114 | 61 11 16 | 3.00E-06 |
|  | L inferior frontal (BA 47) | 78 | -53 24 -3 | 1.79E-04 |
|  | R preSMA (BA 6) | 131 | 6 16 53 | 3.20E-05 |
|  | L premotor (BA 6) | 108 | -46 1 49 | 4.10E-05 |
|  | R pre/postcentral (BA 3,4) | 120 | 44 -12 61 | 5.00E-06 |
|  | L postcentral (BA 3) | 88 | -39 -23 54 | 4.10E-05 |
|  | L superior parietal (BA 7) | 122 | -28 -60 64 | 1.50E-05 |
|  | R fusiform (BA 37) | 79 | 41 -62 -14 | 2.00E-05 |
|  | L middle/inferior occipital (BA 18) | 200 | -36 -91 7 | 6.40E-05 |
|  | R middle/inferior occipital (BA 18,19) | 376 | 35 -88 6 | 1.60E-05 |
|  | L cuneus (BA 18) | 132 | -12 -70 11 | 2.00E-06 |
|  | B lingual, cuneus (BA 18) | 121 | 0 -78 6 | 2.10E-05 |
| **Parietal-Occipital** |  |  |  |  |
| mPC (BA 31) | L inferior frontal (BA 45) | 127 | -67 19 19 | 3.3502E-07 |
|  | L inferior frontal (BA 47) | 484 | -53 39 -5 | 1.3827E-07 |
|  | L middle temporal (BA 21) | 80 | -66 -20 -12 | 6.00E-06 |
|  | R angular gyrus (BA 39) | 145 | 53 -61 38 | 2.50E-05 |
| L IP (BA 40) | L medial superior frontal (BA 9) | 183 | -7 48 27 | 1.00E-06 |
| L AG (BA 39) | R temporal pole (BA 36,38) | 183 | 28 2 -41 | 2.0295E-09 |
|  | L temporal pole (BA 36,38) | 109 | -30 5 -43 | 3.00E-06 |
| R cuneus (BA 19) | L middle frontal (BA 10) | 209 | -14 69 35 | 7.451E-07 |
|  | L medial dorsal thalamus | 104 | -8 -16 8 | 1.2358E-08 |
| **Temporal** |  |  |  |  |
| L IT (BA 20) | R superior/middle frontal (BA 10) | 105 | 41 61 15 | 1.35E-04 |
|  | R orbitofrontal (BA 11) | 216 | 42 52 -14 | 2.00E-06 |
|  | L inferior frontal (BA 44,45) | 110 | - 59 18 13 | 2.60E-05 |
|  | R inferior frontal (BA 9) | 147 | 49 13 29 | 3.00E-05 |
|  | R parahippocampus (BA 37) | 41 | 26 -48 -5 | 1.00E-06 |
|  | L cuneus (BA 19) | 246 | -25 -89 40 | 1.10E-05 |
|  | R cuneus (BA 19) | 77 | 29 -91 40 | 3.00E-06 |
|  | R globus pallidus | 35 | 17 3 1 | 2.00E-06 |
| L aMT (BA 21) | R media superior frontal (BA8) | 90 | 7 39 45 | 1.60E-05 |
|  | L middle frontal (BA 9) | 177 | -46 22 34 | 2.20E-05 |
|  | L superior parietal (BA 7) | 113 | -34 -63 55 | 2.50E-05 |
| R aMT (BA 21) | L inferior temporal (BA 20,37) | 106 | -49 -51 -9 | 2.00E-06 |
| L PH | L precuneus (BA 7) | 123 | -37 -78 41 | 1.70E-05 |
|  | B medial dorsal thalamus | 77 | 1 -10 15 | 2.00E-06 |

**^†^**Montreal Neurological Institute (MNI) brain atlas coordinates.

^‡^ Tabled p values are uncorrected. All p values remained significant after FDR adjustment (p < .001) for 90 PPI features that showed group differences (uncorrected).

B=bilateral hemispheres; L=left hemisphere; R=right hemisphere. AG = angular gyrus; aMT = anterior middle temporal; IP = inferior parietal; IT = inferior temporal; mPC = medial posterior cingulate; PH= parahippocampus; preSMA = pre-supplementary motor area; SF = superior frontal
