# Supplementary material for: Semantic Recollection in Parkinson’s Disease: Functional Reconfiguration and MAPT Variants
Source: Front Aging Neurosci. 2021 Sep 20;13:727057. doi: 10.3389/fnagi.2021.727057 (PMC8489380; doi:10.3389/fnagi.2021.727057)
Supplement: Supplementary file 5 [file Table_5.docx]

**Supplementary Table 5**. Fame-modulated couplings that were stronger in the PD than in the control group.

| **Seed** | **Region (Brodmann area)** | **Voxels** | **MNI^†^** | **p value^‡^** |
| --- | --- | --- | --- | --- |
| **Frontal** | **PD > Control** |  |  |  |
| L mSF (BA 9) | R paracentral (BA 5) | 102 | 10 -33 76 | 3.00E-06 |
|  | L posterior cingulate (BA 31) | 108 | -24 -24 45 | 7.381E-10 |
|  | R inferior parietal (BA 40) | 84 | 51 -48 61 | 7.70E-05 |
| L mSF (BA 10) | R mSFG (BA 10) | 93 | 19 47 11 | 1.8424E-07 |
|  | L caudate body | 36 | -5 3 11 | 1.10E-05 |
| R mSF (BA 11) | L SMA (BA 6) | 80 | -3 -14 79 | 8.10E-05 |
|  | L precuneus/SPL (BA 7) | 78 | -17 -49 67 | 2.00E-05 |
| R mSF (BA 9) | R middle occipital (BA 18) | 78 | 31 -106 9 | 9.597E-07 |
| R mF (BA 10) | R SMA (BA 6) | 122 | 16 -18 83 | 2.00E-06 |
|  | L precuneus | 108 | -7 -77 58 | 7.30E-05 |
|  | L angular gyrus (BA 39) | 76 | -52 -77 23 | 3.70E-05 |
|  | R angular gyrus (BA 39) | 100 | 55 -77 21 | 4.7511E-07 |
|  | R fusiform | 88 | 25 -96 -11 | 1.11E-04 |
| L AC (BA 32) | L caudate body | 74 | -3 -1 21 | 3.6789E-08 |
| **Parietal-Occipital** |  |  |  |  |
| R precuneus | R inferior parietal (BA 40) | 115 | 59 -31 46 | 1.20E-04 |
| L AG (BA 39) | B isthmus cingulate (BA 29) | 93 | 4 -49 0 | 1.4794E-07 |
|  | B caudate body | 83 | 2 5 10 | 6.00E-06 |
|  | R caudate body | 49 | 18 13 17 | 4.00E-06 |
| **Temporal** |  |  |  |  |
| L IT (BA 20) | L caudate body | 79 | -3 6 12 | 1.00E-06 |
|  | L putamen | 47 | -22 10 -9 | 1.10E-05 |
| R IT (BA 20) | R media frontal, preSMA (BA 6,9) | 100 | 4 38 32 | 2.30E-05 |
|  | R inferior parietal (BA 40) | 172 | 53 -48 53 | 5.00E-06 |
| R PH | R orbitofrontal (BA 10) | 85 | 17 40 -11 | 1.0269E-07 |
| **Subcortical** |  |  |  |  |
| L caudate | R superior frontal (BA 8) | 115 | 29 32 50 | 2.10E-05 |
|  | R postcentral (BA 3) | 201 | 49 -19 60 | 5.00E-06 |
|  | R postcentral (BA 2) | 78 | 53 -21 44 | 2.80E-05 |
|  | R precuneus (BA 7) | 101 | 13 -56 65 | 1.70E-05 |
|  | R transverse temporal (BA 42) | 309 | 66 -13 18 | 1.5518E-07 |
|  | L transverse temporal (BA 42) | 195 | -62 -12 12 | 1.00E-06 |
|  | R superior temporal (BA 22) | 169 | 61 13 -8 | 6.4897E-07 |
|  | R medial temporal (BA 21) | 126 | 62 -51 -1 | 8.00E-06 |
|  | R middle temporal (BA 37) | 77 | 44 -59 -3 | 2.00E-06 |

^‡^ Tabled p values are uncorrected. All p values remained significant after FDR adjustment (p < .001) for 81 PPI features that showed group differences (uncorrected).

**^†^**Montreal Neurological Institute (MNI) brain atlas coordinates.

L=left hemisphere; R=right hemisphere. AC = anterior cingulate; AG = angular gyrus; IT = inferior temporal; mF = medial frontal; mSF = medial superior frontal; ; PH = parahippocampus; preSMA = pre-supplementary motor area; SMA = supplementary motor area; ACC needs definition
