# Supplementary material for: Semantic Recollection in Parkinson’s Disease: Functional Reconfiguration and MAPT Variants
Source: Front Aging Neurosci. 2021 Sep 20;13:727057. doi: 10.3389/fnagi.2021.727057 (PMC8489380; doi:10.3389/fnagi.2021.727057)
Supplement: Supplementary file 6 [file Table_6.docx]

**Supplementary Table 6**. Fame-modulated couplings that did not differ between the PD and the control groups.

| **Seed** | **Region (Brodmann area)** | **Voxels** | **MNI^†^** | **p value^‡^** |
| --- | --- | --- | --- | --- |
| **Frontal** |  |  |  |  |
| L SF (BA 10) | R middle frontal (BA 9) | 92 | 35 43 43 | 1.00E-03 |
|  | R middle frontal (BA 6) | 129 | 44 2 52 | 1.14E-04 |
|  | R premotor (BA 6) | 168 | 38 -14 69 | 7.00E-05 |
|  | B SMA (BA 6) | 127 | 0 -18 58 | 2.10E-05 |
|  | L paracentral (BA 5) | 111 | -32 -45 61 | 7.40E-05 |
|  | L inferior parietal, postcentral (BA 2,40) | 241 | -43 -31 54 | 6.30E-05 |
|  | L precuneus (BA 7) | 1023 | -10 -77 47 | 2.00E-06 |
|  | R precuneus (BA 7) | 1176 | 13 -63 64 | 1.1122E-07 |
|  | L inferior parietal (BA 40) | 98 | -52 -35 24 | 5.00E-06 |
|  | L temporal pole (BA 38) | 184 | -21 3 -34 | 2.562E-09 |
|  | L superior temporal (BA 22) | 81 | -63 -45 17 | 3.32E-04 |
|  | R superior/middle temporal (BA 21,22) | 81 | 66 -4 -8 | 2.00E-06 |
| R mF (BA 10) | R inferior frontal (BA 45) | 96 | 57 25 20 | 5.60E-05 |
| L mSF (BA 10) | R middle occipital (BA 19) | 99 | 37 -87 18 | 1.00E-03 |
| L AC (BA 32) | L middle occipital (BA 19) | 247 | -29 -85 23 | 6.70E-05 |
| **Parietal-Occipital** |  |  |  |  |
| mPC (BA 31) | L inferior frontal (BA 9,44) | 89 | -53 9 25 | 1.00E-05 |
|  | L inferior frontal (BA 9) | 82 | -46 4 35 | 2.60E-05 |
|  | R inferior frontal (BA 46) | 80 | 56 25 15 | 7.70E-05 |
|  | L anterior cingulate (BA 32) | 135 | -8 14 41 | 6.9531E-07 |
|  | L pre/postcentral (BA 3,4) | 240 | -39 -22 55 | 2.2211E-07 |
| R precuneus | R cuneus (BA 18) | 82 | 24 -85 27 | 1.26E-04 |
| L IP (BA 40) | L inferior frontal insula (BA 13,47) | 212 | -38 28 1 | 4.5795E-07 |
|  | L inferior frontal (BA 45) | 100 | -52 11 26 | 1.21E-04 |
|  | R inferior frontal (BA 46) | 83 | 48 28 19 | 4.62E-04 |
|  | R putamen | 67 | 21 7 1 | 1.1621E-08 |
| L AG (BA 39) | R anterior cingulate (BA 32) | 128 | 4 19 35 | 8.1121E-07 |
|  | B medial superior frontal, preSMA (BA 6,8) | 439 | 1 18 53 | 8.5159E-07 |
|  | L middle/inferior frontal (BA 9,46) | 258 | -53 17 25 | 8.7244E-07 |
|  | R inferior frontal (BA 45,47) | 823 | 42 27 6 | 4.8328E-08 |
|  | L inferior frontal, insula (BA 13,47) | 236 | -39 27 -1 | 1.0543E-07 |
|  | L premotor (BA 6) | 134 | -45 -3 53 | 4.50E-05 |
|  | L SMA (BA 6) | 107 | -5 0 66 | 5.00E-05 |
|  | R premotor (BA 6) | 492 | 52 -3 47 | 7.1955E-07 |
|  | R superior parietal (BA 7) | 143 | 30 -62 56 | 3.80E-05 |
|  | L precuneus, cuneus (7,19) | 728 | -29 -89 23 | 3.00E-06 |
|  | L middle temporal (BA 22) | 86 | -54 -43 2 | 1.7432E-07 |
|  | B cuneus (BA 18) | 258 | -4 -72 13 | 1.00E-06 |
|  | R middle occipital (BA 19) | 464 | 37 -86 17 | 8.00E-06 |
|  | L lingual (BA 19) | 85 | -24 -73 -6 | 1.00E-06 |
| R cuneus (BA 19) | L preSMA (BA 6) | 77 | -4 14 61 | 2.67E-04 |
|  | R anterior parahippocampus | 35 | 27 -7 -22 | 5.80E-05 |
| **Temporal** |  |  |  |  |
| L aMT (BA 21) | L inferior frontal (BA 9) | 243 | -47 15 29 | 2.80E-05 |
|  | R inferior frontal (BA 47) | 201 | 35 28 2 | 3.00E-06 |
|  | R inferior frontal (BA 46) | 179 | 45 25 24 | 1.90E-05 |
|  | B preSMA (BA 6) | 228 | -4 18 54 | 2.00E-06 |
| L IT (BA 20) | B anterior cingulate, R preSMA, R medial superior frontal (BA 6,8,32) | 754 | 2 21 45 | 1.7164E-07 |
|  | R middle frontal (BA 9) | 293 | 39 41 31 | 2.00E-06 |
|  | R middle frontal (BA 9) | 141 | 42 10 35 | 2.60E-05 |
|  | L inferior frontal (BA 9) | 310 | -54 10 24 | 3.00E-06 |
|  | R inferior frontal (BA 47) | 584 | 37 27 0 | 4.2581E-08 |
|  | L middle frontal (BA 6) | 242 | -51 0 52 | 1.00E-05 |
|  | L SMA (BA 6) | 436 | -7 -1 64 | 4.2335E-07 |
|  | L postcentral (BA 2) | 379 | -42 -27 49 | 1.8438E-07 |
|  | R paracentral (BA 5) | 93 | 4 -30 55 | 8.00E-06 |
|  | L superior parietal (BA 7) | 99 | -27 -58 51 | 2.80E-05 |
|  | L precuneus (BA 7) | 227 | -6 -72 47 | 9.00E-06 |
|  | R precuneus (BA 7) | 310 | -4 -69 21 | 3.0013E-07 |
|  | R posterior cingulate (BA 30) | 104 | 20 -57 3 | 1.00E-06 |
|  | R inferior parietal (BA 40) | 108 | 55 -28 47 | 1.50E-05 |
|  | R cuneus (BA 18) | 79 | 12 -76 11 | 2.35E-04 |
|  | R medial dorsal thalamus | 90 | 7 -22 -3 | 3.1602E-08 |
| R IT (BA 20) | L cuneus (BA 18) | 94 | -3 -82 10 | 7.70E-05 |
| **Subcortical** |  |  |  |  |
| R caudate | B anterior cingulate (BA 32, 24) | 201 | 7 20 38 | 6.4169E-07 |

^‡^ Tabled p values are uncorrected. All p values remained significant after FDR adjustment (p < .001) for 77 PPI features that exhibited stronger connectivity for famous than unfamiliar names in both groups (uncorrected).

**^†^**Montreal Neurological Institute (MNI) brain atlas coordinates.

B=bilateral hemispheres; L=left hemisphere; R=right hemisphere. AC = anterior cingulate; AG = angular gyrus;; aMT = anterior middle temporal;;; IP = inferior parietal; IT = inferior temporal; mF = medial frontal; mPC = medial posterior cingulate; mSF = medial superior frontal;; preSMA = pre-supplementary motor area; SF = superior frontal; SMA = supplementary motor area
